# SnSe_1‐x_S_x_ Alloys: Anisotropic Van der Waals Semiconductors with Tunable Bandgaps

**DOI:** 10.1002/smll.202508578

**Published:** 2025-11-27

**Authors:** Peter Sutter, Alexei Barinov, Hannu‐Pekka Komsa, Pramod Ghimire, Lijun Wu, Yimei Zhu, Kim Kisslinger, Eli Sutter

**Affiliations:** ^1^ Department of Electrical & Computer Engineering University of Nebraska‐Lincoln Lincoln NE 68588 USA; ^2^ Elettra Sincrotrone Trieste SCpA Basovizza Trieste 34149 Italy; ^3^ Microelectronics Research Unit University of Oulu Oulu FI‐90014 Finland; ^4^ Department of Mechanical & Materials Engineering University of Nebraska‐Lincoln Lincoln NE 68588 USA; ^5^ Condensed Matter Physics and Materials Science Department Brookhaven National Laboratory Upton NY 11973 USA; ^6^ Center for Functional Nanomaterials Brookhaven National Laboratory Upton NY 11973 USA; ^7^ Nebraska Center for Materials & Nanoscience University of Nebraska‐Lincoln Lincoln NE 68588 USA

**Keywords:** anisotropic electronic structure, bandgap, tin monochalcogenides, valleytronics, Van der Waals alloys

## Abstract

Alloying is one of the main tools of bandgap engineering, allowing the tuning of crystal structure, lattice parameters, and electronic structure of 3D and 2D/layered semiconductors. Among the latter, it can play a key role in tailoring the properties of tin monochalcogenides, a class of van der Waals semiconductors of interest for optoelectronics, thermoelectrics, ferroelectrics, and valleytronics. Here, the study investigates the synthesis and properties of large flakes of the anion substitution alloys SnSe_1‐x_S_x_. Alloy flakes across a wide range of compositions are obtained systematically by repeated growth from the same mixed (SnS, SnSe) powder precursor. Combined experiment and theory show full miscibility for all compositions, along with tunable lattice constants, bandgaps, and vibrational modes. Atomic resolution imaging demonstrates the accumulation of S and Se in alternating layers in the SnSe_1‐x_S_x_ unit cell, attributed to growth kinetics. Polarized Raman spectroscopy confirms anisotropic vibrational modes; the calculated and measured band structure shows systematic changes in the band edge energies and anisotropic electronic structure due to the anisotropic in‐plane lattice of the monochalcogenides. Cathodoluminescence, finally, indicates that a unique configuration of two non‐degenerate, direct valleys along orthogonal *k*‐space directions persists all the way from SnS to SnSe, making SnSe_1‐x_S_x_ alloys interesting for valleytronics.

## Graphical abstract

Consecutive growths from a mixed (SnS‐SnSe) powder precursor produced series of samples of large, single‐crystalline SnSe1‐xSx alloy flakes with systematically varying composition. Advanced experiments on such samples, combined with theoretical calculations, enabled the comprehensive investigation of the crystal structure, vibrational modes, band structure, and optoelectronic properties of anisotropic SnSe1‐xSx alloys, showing promising properties for optoelectronics, ferroelectrics, thermoelectrics, and valleytronics.

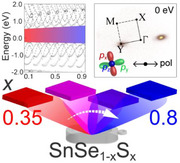

## 1 Introduction

Alloying has long been a mainstay of band structure engineering and it continues to be an important tool for tuning structure, electronic, and optical properties of two‐dimensional (2D) and layered van der Waals semiconductors. Among such layered crystals, the monochalcogenides SnS and SnSe are of particular interest due to several exceptional characteristics, including anisotropic electronic,^[^ ^1^ ^]^ optical,^[^ ^2^ ^]^ and vibrational properties,^[^ ^3^, ^4^ ^]^ outstanding thermoelectric performance,^[^ ^1^, ^5^ ^]^ as well as in‐plane ferroelectricity,^[^ ^6^, ^7^, ^8^, ^9^, ^10^, ^11^, ^12^, ^13^ ^]^ among others. SnSe_1−x_S_x_ alloys, predicted to be stable as random solid solutions across the entire range of composition (*x*) at room temperature and above,^[^ ^14^, ^15^ ^]^ have been employed to enhance thermoelectric properties^[^ ^16^, ^17^, ^18^, ^19^, ^20^ ^]^ and tune structure and functional characteristics.^[^ ^15^, ^20^, ^21^, ^22^, ^23^ ^]^ Finally, the anisotropic band structure comprising direct electronic transitions along the perpendicular Γ‐X and Γ‐Y directions in reciprocal space, i.e., the presence of non‐degenerate X‐ and Y‐valleys that can be addressed via linearly polarized light and read out optically^[^ ^24^ ^]^ and possibly electrically,^[^ ^25^ ^]^ has created interest in SnS as a semiconductor for valleytronics.^[^ ^26^ ^]^ In this context, the ability to tune the bandgaps at both valleys via SnSe_1−x_S_x_ alloying could further support such applications, e.g., by bringing a valley into resonance with a particular excitation source.

To access SnSe_1−x_S_x_ alloys two major routes are available, namely bulk crystal growth combined with exfoliation to obtain layered crystal flakes, or direct synthesis of van der Waals flakes on suitable substrates. For tin monochalcogenides, the preparation of high‐quality bulk crystals is complex and time‐consuming, with synthesis runs typically lasting several days. Furthermore, exfoliation makes it difficult to control the shape, lateral size, and thickness of the resulting flakes. Direct synthesis, for example by vapor transport or solution methods, has been widely employed to obtain flakes of the endpoint compounds SnSe^[^ ^9^, ^12^, ^27^, ^28^ ^]^ and SnS,^[^ ^7^, ^11^, ^28^, ^29^ ^]^ as well as heterostructures between them.^[^ ^30^, ^31^ ^]^ However, the formation of SnSe‐SnS ternary flakes has remained limited to two compositions, namely SnS_0.5_Se_0.5_^[^ ^32^, ^33^ ^]^ and SnS_0.25_Se_0.75_.^[^ ^34^ ^]^ In recent work, we demonstrated a robust vapor transport process that allows the rational synthesis of large, thin (few‐layer) SnSe_1−x_S_x_ alloy flakes whose composition is set by the molar fractions of SnS and SnSe in a mixed powder precursor.^[^ ^12^ ^]^ In this process, the use of sufficiently large amounts of precursor (typically ≈0.4 g) enabled the growth of alloy flakes with fixed composition in several consecutive synthesis runs, reusing the same powder mixture.

Here, we explore the opposite regime, namely the use of small amounts of precursors of mixed tin monochalcogenides for the consecutive synthesis of alloy flakes of different composition. We show that minor differences in vapor pressure between the monochalcogenides SnS and SnSe can be exploited to controllably access a series of different anion‐substitution (SnSe_1‐x_S_x_) alloys with progressively increasing sulfur content *x* via successive growth runs using the same mixed SnS‐SnSe precursor. Characterization by optical microscopy, (scanning) transmission electron microscopy ((S))TEM) and electron diffraction, optical absorption, and Raman spectroscopy show that the resulting flakes with typical sizes of ≈20 µm across the entire composition range between SnS and SnSe are high‐quality single crystals whose properties (e.g., the fundamental bandgaps) interpolate between the endpoint compounds. Whereas density functional theory (DFT) predicts that random alloys are stable across the entire composition range, atomic‐resolution STEM on cross‐sections of alloy samples shows a tendency toward enrichment of S and Se in alternating layers of the van der Waals crystals. Calculations of the effective band structure of the alloys are compared with the measured valence band structure obtained by angle‐resolved photoelectron spectroscopy (ARPES), showing a good overall match while also reflecting the pronounced in‐plane structural and electronic anisotropy of the monochalcogenide alloys. Cathodoluminescence spectroscopy on individual SnSe_1‐x_S_x_ alloy flakes shows light emission dominated by direct transitions between band edges along perpendicular *k*‐space axes, which, in combination with calculations, confirms the unusual electronic configuration of non‐degenerate X‐ and Y‐valleys across the entire composition range from SnSe to SnS. Our work harnesses a facile route for the synthesis of high‐quality flakes of SnSe_1‐x_S_x_ alloys with systematically changing composition to establish the properties of a family of van der Waals semiconductors that are promising for a wide range of applications, including optoelectronics, thermoelectrics, ferroelectricity, and unconventional valleytronics.

## 2 Results and Discussion

Series of SnSe_1‐x_S_x_ alloys were obtained by vapor transport from a mixed (SnS, SnSe) precursor on mica van der Waals substrates (see Experimental Section for details). The precursors evaporate in complete formula units, i.e., liberate SnS or SnSe molecules into the vapor phase upon heating.^[^ ^29^ ^]^ By using a small amount of the mixed precursor powders (typically ≈0.1 g), finely ground, filled into a single crucible, and heated to the growth temperature (580 °C), small differences between the vapor pressures of SnS and SnSe cause a progressive change in the abundance of sulfide and selenide in consecutive growth runs (with continued use of the changing precursor mixture). For example, a mixture with an initial SnSe:SnS ratio of 2:1 first yields Se‐rich SnSe_1‐x_S_x_ alloys (with sulfur content *x* ≈ 0.35), followed by alloys with increasing sulfur content in subsequent growth runs up to *x* ≈ 0.80 in the 8th growth from the same powder.

**Figure 1:**
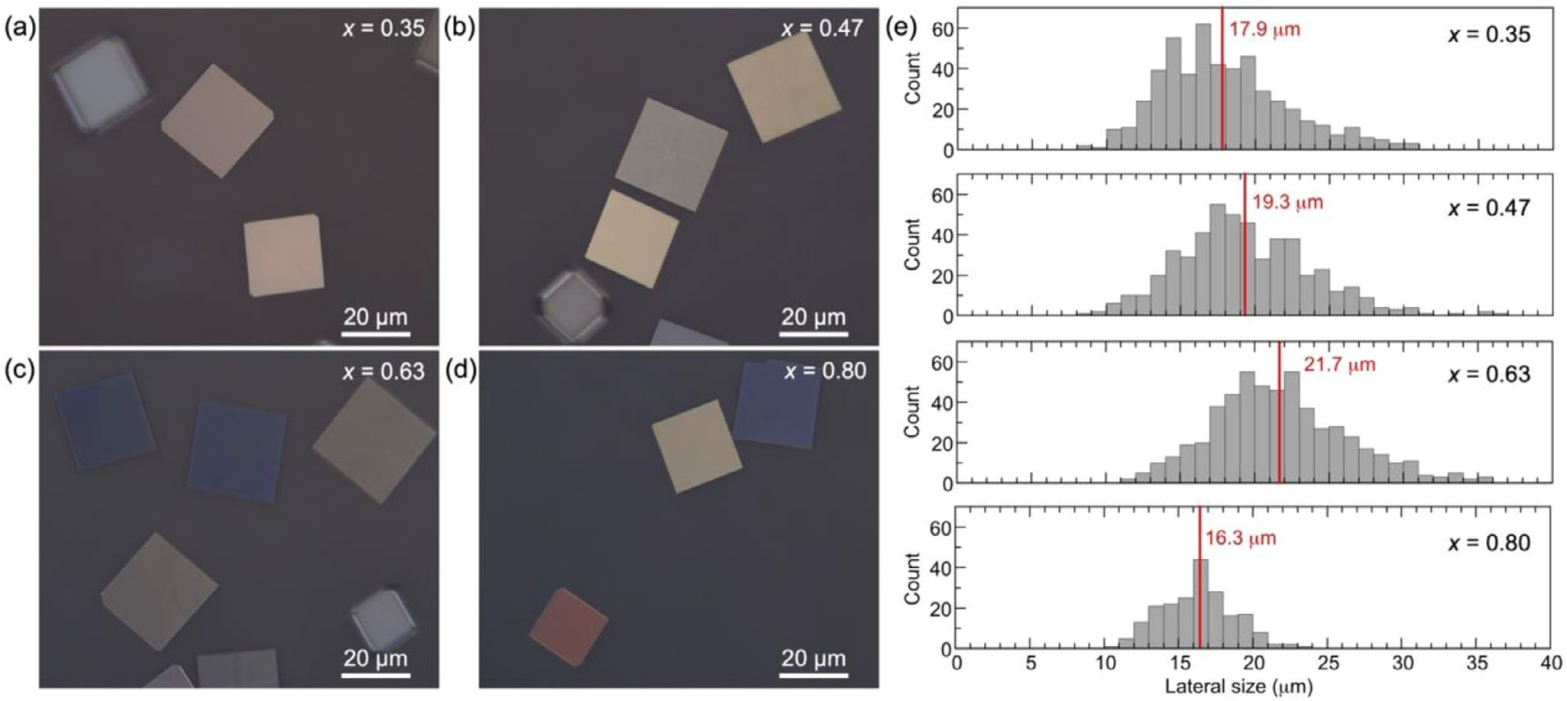
Polarized optical microscopy of characteristic SnSe_1‐x_S_x_ alloy flakes obtained by successive growth from the same precursor powder. a–d) Flakes with sulfur content *x* = 0.35 (a), *x* = 0.47 (b), *x* = 0.63 (c), and *x* = 0.80 (d). Different colors are the result of a combination of varying flake thickness and different orientations of the flakes with respect to the axis of the polarization of the incident light. e) Histograms of the lateral flake size distributions for the samples shown in (a–d). Vertical lines indicate the mean size. Note that the sample with *x* = 0.80 has been subjected to multiple flake transfers. Hence, only a reduced number of flakes could be measured for this sample, and the size distribution may be affected as well.

**Figure 2:**
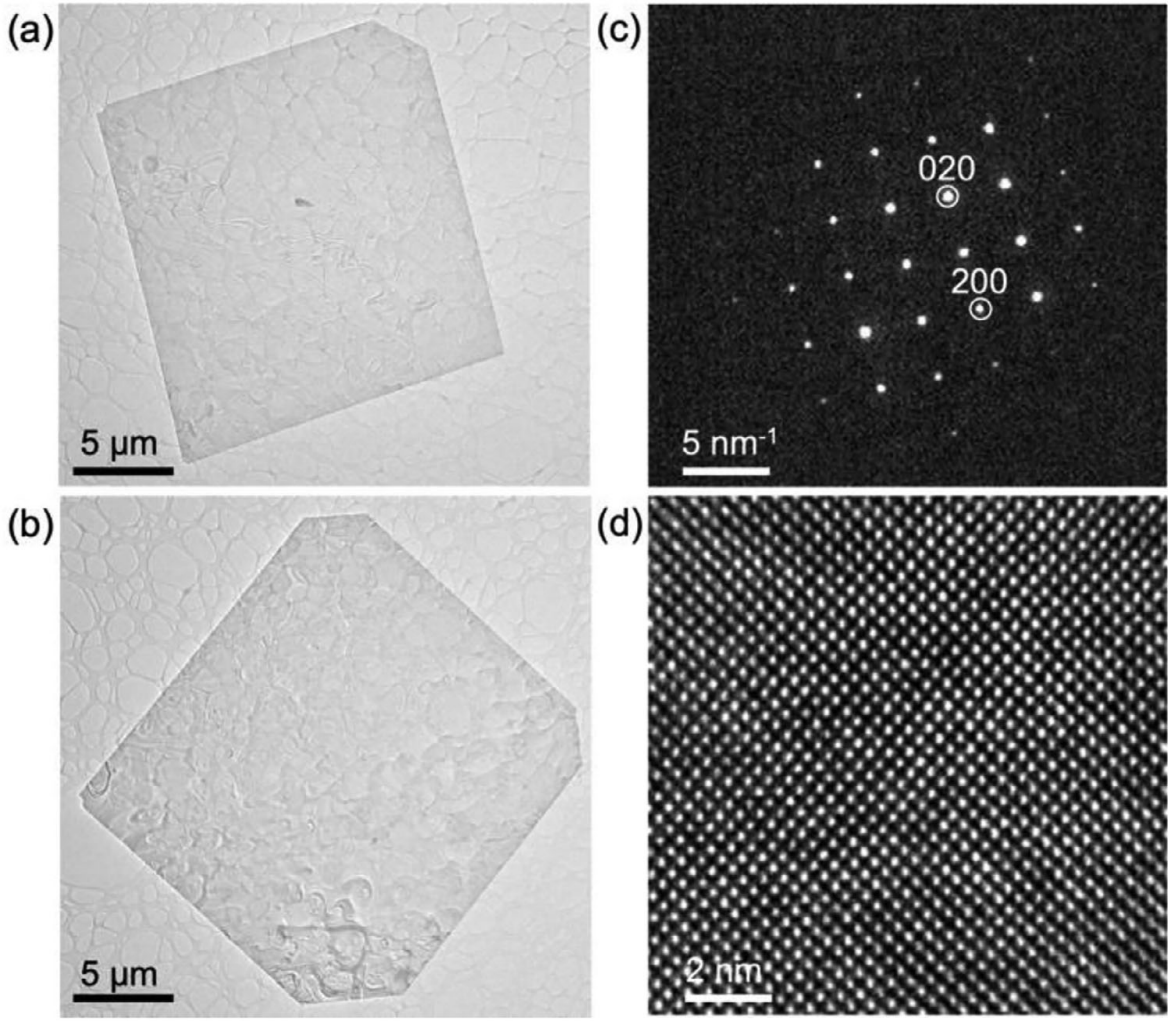
Transmission electron microscopy and diffraction of characteristic SnSe_1‐x_S_x_ alloy flakes. a,b) TEM images of characteristic few‐layer SnSe_1‐x_S_x_ flakes (*x* = 0.80) transferred to lacey carbon support. c) Nanobeam electron diffraction pattern obtained on the flake shown in (a). d) High‐resolution TEM image obtained on the flake shown in (b).

Optical microscopy demonstrates that the resulting alloy flakes have well‐defined shapes bounded by flat basal facets and long, straight edge facets for all compositions (**Figure** **1**a–d). A statistical analysis shows mean lateral sizes ranging between 16 µm (for *x* = 0.80) and 22 µm (for *x* = 0.63), without substantial changes over several growths from the same precursor (Figure 1e). The SnSe_1‐x_S_x_ alloy flakes were further characterized by TEM and electron diffraction. TEM imaging confirms a uniform morphology bounded by (001) top/bottom and extended side facets (**Figure** **2**a,b) identified by diffraction analysis as {110} facets (Figure 2c), similar to flakes of the endpoint compounds SnS and SnSe obtained by vapor transport growth. Electron diffraction (Figure 2c) and high‐resolution TEM imaging (Figure 2d) demonstrate that the flakes are high‐quality single crystals adopting the orthorhombic structure (space group *Pnma*) characteristic of layered tin monochalcogenides.

Among the primary goals pursued by alloying of semiconductors are engineering of the crystal structure (e.g., to systematically vary the lattice constants) and of the electronic band structure (e.g., to tune the fundamental bandgap, *E*_g_). We combined experiments and theoretical calculations to establish the dependence of the lattice parameters and of the band structure on the composition of SnSe_1‐x_S_x_ alloy flakes.

Both density functional theory (DFT) and cluster expansion (CE) calculations show small mixing energies (below ≈15 meV per formula unit), indicating that SnSe_1‐x_S_x_ random alloys should be stable across the entire composition range at room temperature (Figure S1, Supporting Information). The properties of random alloys were calculated for special quasi‐random structures (SQS) constructed using the ATAT package (see Experimental Section). Computed and measured lattice constants are given in Figure S2 and Table S1 (Supporting Information). Tin monochalcogenides are anisotropic orthorhombic layered crystals with in‐plane lattice constants *a* and *b* defined by covalent intralayer bonding, and an out‐of‐plane unit cell size *c* that spans two individual layers. Calculations for all lattice constants show a linear dependence on composition, *x*, i.e., the lattice parameters follow Vegard's law.

**Figure 3:**
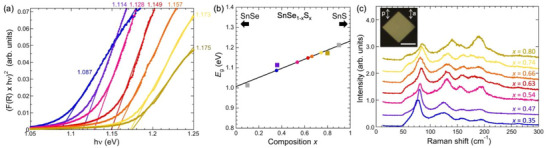
Optical absorption, bandgaps, and Raman spectroscopy of SnSe_1‐x_S_x_ alloy flakes. a) Kubelka–Munk analysis of the optical absorption measurements of seven SnSe_1‐x_S_x_ alloys grown consecutively from the same mixed (SnS, SnSe) powder precursor. Annotations give the bandgaps determined from the intercepts along the energy axis. b) Composition‐dependent bandgaps determined from the absorption measurements (colored symbols), based on (composition‐bandgap) calibration using selected alloys whose compositions were measured by EDS (see Figures S4–S6, Supporting Information). c) Raman spectra of individual flakes of the alloys shown in (a), with sulfur content *x* determined from the bandgaps in (b). Parallel polarization configuration geometry, as shown in the inset. “p”: incident light polarizer axis; “a”: scattered light analyzer axis. Scale bar: 10 µm.

To assess the optical and vibrational properties as a function of composition, spectrophotometry and Raman spectroscopy were performed. The calculated bandgaps show a linear dependence of *E*_g_ on sulfur content, *x* (see Figure S3, Supporting Information). Optical absorption measurements, carried out in diffuse reflectance geometry, were analyzed using the Kubelka‐Munk formalism to determine *E*_g_ on ensembles of SnSe_1‐x_S_x_ alloy flakes. Results for seven alloys grown consecutively from the same precursor are shown in **Figure** **3**a. To analyze the composition‐dependent bandgaps, measurements of both the composition and optical gap were carried out for four alloys (Figure 3b, square symbols) to obtain the linear dependence of *E*_g_(*x*). This relationship, in turn, was used to determine the composition of the remaining alloy samples. A summary of this analysis, shown in Figure 3b, confirms that the sequential growth of alloys from the progressively changing precursor mixture can access a wide range of SnSe_1‐x_S_x_ alloy compositions, including sulfur contents in the range 0.35 ≤ *x* ≤ 0.8. Our experiments indicate that compositions outside of this window, i.e., richer in either S or Se, will require different initial stoichiometries of the precursor mixture.

The progressively increasing sulfur content in the alloy samples represented in Figure 3a,b is also reflected in their vibrational properties. Figure 3c summarizes Raman spectra obtained from these alloys. The anisotropic crystal structure across the entire range of tin monochalcogenides manifests itself in anisotropic vibrational properties where the intensities of the different modes vary with angle between the polarization of the incident laser light and the major in‐plane crystal axes (*a*, *b*). Therefore, to facilitate the comparison of Raman spectra from SnSe_1‐x_S_x_ alloys with different composition, measurements were carried out on flakes whose edge facets allowed a clear identification of the *a*‐ and *b*‐axes (and which therefore could be measured in the same orientation). Spectra obtained in this way (for parallel polarizer and analyzer axes, see inset of Figure 3c) show systematic shifts due to the different mass of S and Se in the energies of peaks corresponding to a single Raman‐active mode, notably the *A*_g_^2^ peak, which shifts from ≈70 cm^−1^ in SnSe to ≈90 cm^−1^ in SnS.

**Figure 4:**
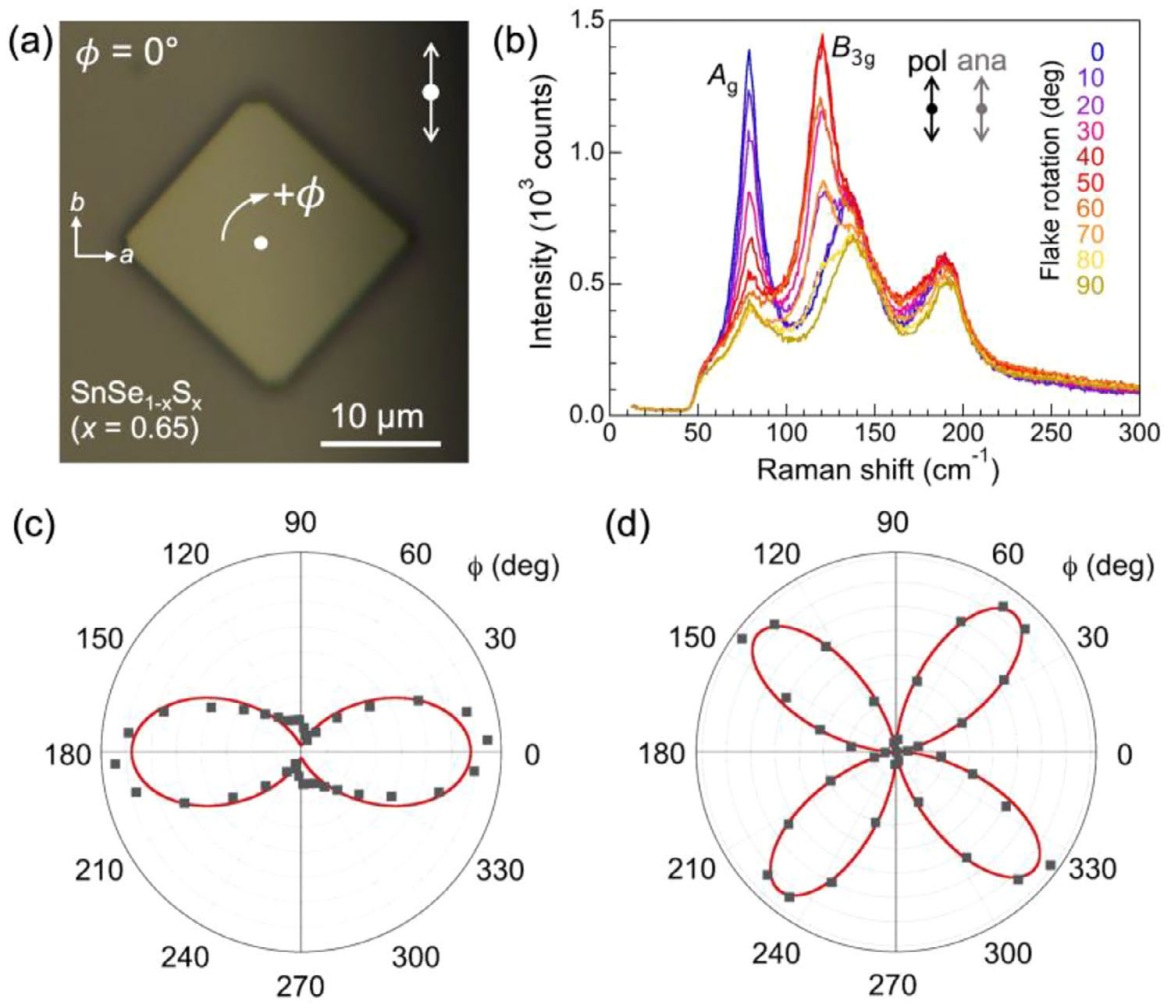
Anisotropic vibrational modes of SnSe_1‐x_S_x_ alloy flakes analyzed by polarized Raman spectroscopy. a) Optical microscopy of a characteristic faceted SnSe_1‐x_S_x_ alloy flake (*x* = 0.65). The angle *ϕ* represents the rotation of the flake relative to the polarization axis of the incident light (indicated by a double arrow, upper right). b) Set of Raman spectra obtained in parallel polarization geometry (i.e., with scattered light analyzer parallel to incident light polarizer) for angles *ϕ* ranging from 0 to 90°. c,d) Polar plots of mode intensity as a function of angle *ϕ* for the *A*_g_ mode at 78 cm^−1^ (c), and for the *B*_3g_ mode at 120 cm^−1^ (d). Symbols represent measured Raman intensities, *I*(*ϕ*); red lines are fits *I_A_*_g_(*ϕ*) = (*m* cos^2^ *ϕ* + *n* sin^2^ *ϕ*)^2^ and *I*_B3g_(*ϕ*) = *k* ^2^ (sin2*ϕ*)^2^, respectively.^[^ ^35^ ^].^

The vibrational modes also reflect the structural anisotropy of SnSe_1‐x_S_x_ alloy crystals, as illustrated in polarized Raman measurements shown in **Figure** **4**. The alloy flakes are generally bounded by straight {110} facets and, due to the difference in *a* and *b* in‐plane lattice parameters, adopt a characteristic rhombic shape with angles between adjacent edges from which the major crystal axes can be identified (see Figure 4a). In polarized Raman spectra, measured here in parallel polarization configuration for a SnSe_0.35_S_0.65_ alloy, the intensity of the primary vibrational modes depends systematically on the orientation of the flake, quantified via the angle *ϕ* between the polarization and the *b*‐axis of the flake (Figure 4b). Figure 4c,d illustrate the vibrational anisotropy via polar plots of the intensity *I*(*ϕ*) for the *A*_g_ mode at ≈78 cm^−1^ and the *B*_3g_ mode at ≈120 cm^−1^, respectively. In both cases, the polarization dependence follows the pattern observed for the endpoint compounds SnS,^[^ ^36^ ^]^ SnSe,^[^ ^37^ ^]^ as well as SnSe_0.5_S_0.5_,^[^ ^38^ ^]^ confirming that intermediate SnSe_1‐x_S_x_ alloys host anisotropic vibrational modes.

DFT calculations predict the formation of stable random alloys between the endpoint compounds SnSe and SnS (see Figure S1, Supporting Information). High‐resolution STEM imaging on cross‐sectional samples was used to assess the atomic structure of typical SnSe_1‐x_S_x_ alloy flakes and – combined with electron energy loss spectroscopy (EELS) and mapping – determine the actual distribution of Se and S in the alloy. EDS mapping at low resolution shows a uniform distribution of the constituent elements (Sn, S, Se) across the entire flake (Figure S4, Supporting Information); the spectrum for this sample is quantified to an alloy composition SnSe_0.36_S_0.64_ (Figure S5, Supporting Information).

**Figure 5:**
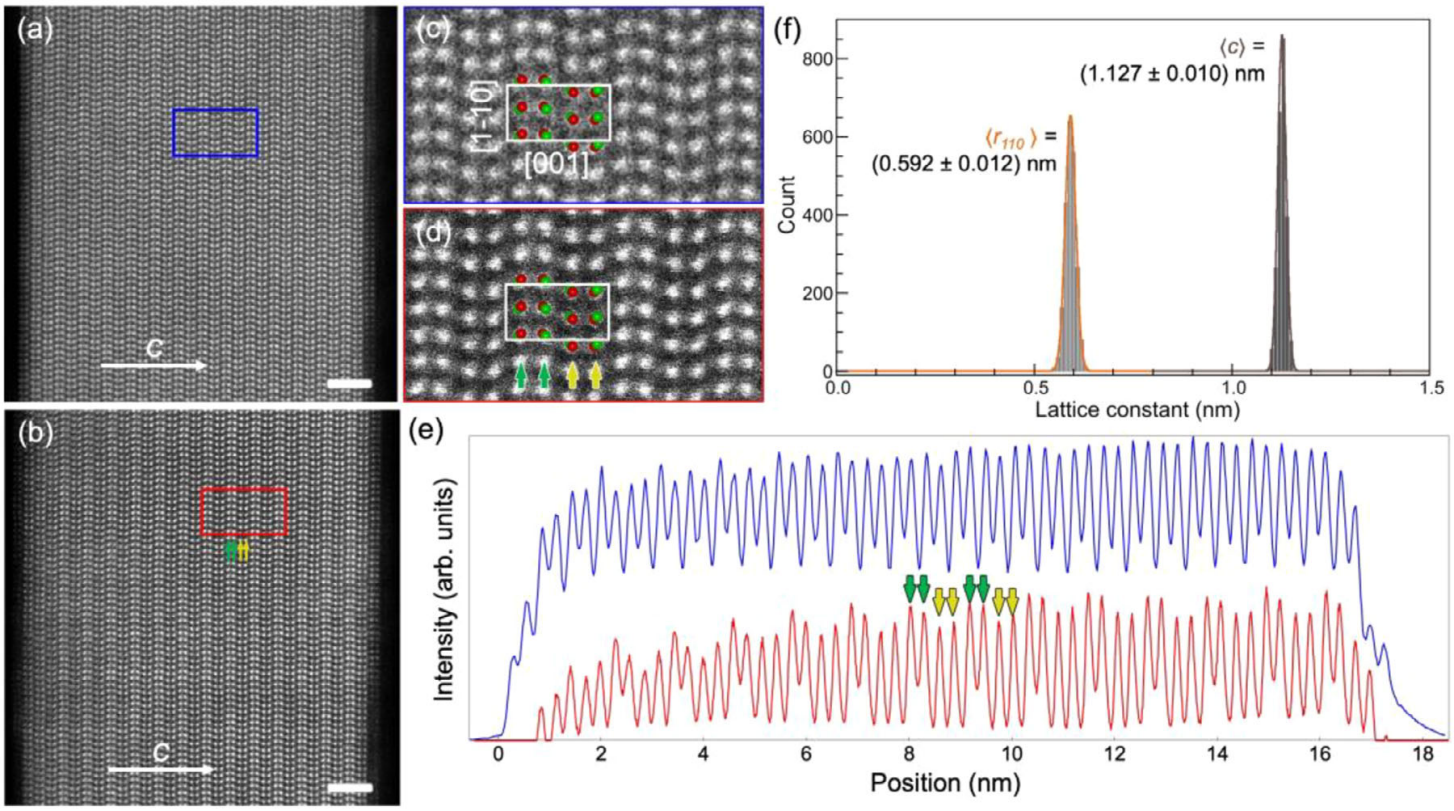
Atomic‐resolution cross‐sectional STEM on SnSe_1‐x_S_x_ alloys. a,b) Two HAADF‐STEM images acquired in different area in a SnSe_1‐x_S_x_ alloy flake (with S content *x* = 0.64). Zone axis: [110]. Scale bar 2 nm. c) Analysis of the unit cell size along the [1 ‐1 0] in‐plane direction and along [0 0 1] (*c*‐axis) based on the atomic positions obtained from HAADF‐STEM images. d,e) Magnified images from the areas marked by blue and red rectangles in (a) and (b), respectively. The [110] projection of the SnSe structure model represents Sn and Se/S atoms by red and green spheres, respectively. White rectangles outline the unit cell. f) Intensity profiles obtained by integrating the image intensity in image (a, blue) and (b, red) vertically. In some areas of the sample (e.g., in (a)), the image intensity distribution is relatively uniform. In most areas, however (e.g., in (b)), the image intensity alternates between bright and darker in adjacent atomic columns along the *c*‐axis. There are two pairs of Sn/Se atoms in a unit cell, as indicated by the green and yellow arrows in the magnified image shown in (d). The image intensity in one pair of Sn/Se columns (green arrow) is stronger than in the other (yellow arrow). This can also be seen in the intensity profiles in (e).

**Figure** **5** summarizes the results of cross‐sectional HAADF‐STEM imaging of the same flake with atomic resolution (zone axis: [110]). Figure 5a,b illustrates the well‐ordered layer structure of the monochalcogenide alloy. No atoms are observed in interstitial positions, i.e., the anion substitution in the alloys involves exclusively lattice positions. Analysis of the in‐plane (*r*_110_) and out‐of‐plane (*c*) unit cell sizes based on atomic positions determined from the STEM images yields narrow histograms for both *r*_110_ and *c* (Figure 5c), consistent with the highly ordered single‐crystalline structure of the layered alloy flakes. While the layers in some areas appear uniform in contrast (Figure 5a,c), most regions show successive layers with alternating brighter and darker contrast (Figure 5b,d). Since the intensity in HAADF‐STEM scales with the average atomic number *Z* and the atomic columns along the [110] axis contain both cations (Sn) and anions (S/Se, see Figure 5d,e), this observation indicates an inhomogeneous distribution of sulfur (*Z* = 16) and selenium (*Z* = 34). HAADF‐STEM intensity profiles along the out‐of‐plane (*c*) direction, shown in Figure 5f, confirm the alternating image contrast between consecutive layers. Since the monochalcogenide unit cell comprises two layers, the observed HAADF‐STEM contrast profile implies different ratios of S:Se in the two layers of the unit cell.

To confirm and quantify the inhomogeneous chalcogen distribution in alternating layers of the synthetic SnSe_1‐x_S_x_ alloy flakes, atomic‐scale chemical analysis was performed using electron energy loss spectroscopy (EELS) in STEM. Figure S7a (Supporting Information), a HAADF‐STEM image of the part of the flake in which this chemical analysis was carried out, again shows alternating bright and darker contrast in consecutive layers (characteristic of most regions of the flake, see Figure 4). The elemental analysis used the *L* edges of S and Se, as well as the *M* edge of Sn (Figure S7b, Supporting Information). Maps of EELS intensity for these edges are shown in Figure S7d–f (Supporting Information), correlated with the corresponding HAADF‐STEM image in Figure S7c (Supporting Information). Figure S7g (Supporting Information), finally, provides line profiles of the EELS and STEM intensities (integrated along the horizontal axis) obtained from these maps. The HAADF‐STEM profile again shows the characteristic alternation of higher and lower image intensity. The Sn *M* signal is uniform across all layers, apart from statistical variations (i.e., noise). In the Se *L* profile, the intensity in consecutive layers alternates between high and low; layers with high Se *L* intensity show low S *L* intensity (and high HAADF‐STEM signal), and *vice versa*. A quantification of the EELS intensity profile along the *c*‐axis (Figure S8, Supporting Information) shows S:Se ratios of 2.3 and 2.8 in consecutive layers of an alloy with average composition SnSe_0.28_S_0.72_. Hence, the atomic‐scale elemental analysis confirms that the synthetic Sn chalcogenides are not random alloys but consistently show small differences in S:Se ratio between the two layers of the unit cell. Given that calculations find a random alloy to be the lowest energy configuration and in view of the layer‐by‐layer growth of the monochalcogenide flakes,^[^ ^11^ ^]^ the alternating S and Se enrichment of the layers appears to result from growth kinetics. Since the precursors evaporate in SnS(e) formula units, a plausible explanation involves an oscillation of the sulfide:selenide ratio at the growth front combined with a pronounced layer‐by‐layer growth in which a new layer expands to cover the entire flake surface before nucleation of the next layer takes place (as observed previously for large SnS flakes grown under the same conditions).^[^ ^11^ ^]^ An initial preferential incorporation of the lower vapor pressure (sulfide) species, for instance, would in turn lead to a small excess of the complementary (selenide) species, which thus becomes enriched in the subsequent layer. A repetition of this process would then yield the observed periodic composition variation between adjacent layers. Given that the oscillatory composition change is strictly observed between consecutive atomic layers in the alloy flakes, alternative explanations, e.g., a periodic variation in the supply of sulfide and selenide precursors from the vapor phase, appear less likely.

**Figure 6:**
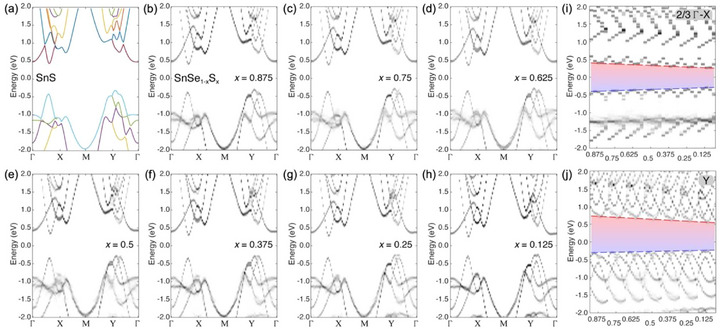
Calculated band structures of SnSe_1‐x_S_x_ alloys. a–h) Band structure of SnS (a), and effective band structures of SnSe_1‐x_S_x_ alloys with sulfur content *x* = 0.875 (b), *x* = 0.75 (c), *x* = 0.625 (d), *x* = 0.5 (e), *x* = 0.375 (f), *x* = 0.25 (g), and *x* = 0.125 (h). i) Evolution of the direct bandgap at 2/3 Γ‐X (X‐valley) with S content *x* of the alloy. j) Evolution of the direct bandgap at Y (Y‐valley) with S content *x* of the alloy.

The electronic structure of the SnSe_1‐x_S_x_ alloys was investigated by DFT calculations and experiments, including angle‐resolved photoelectron spectroscopy (ARPES) and cathodoluminescence spectroscopy. The calculated band structures are summarized in **Figure** **6**. The SnS band structure illustrates the key electronic features of this class of anisotropic van der Waals semiconductors (Figure 6a). While the fundamental bandgap of SnS is indirect, defined by the valence band maximum at Y and the conduction band minimum at 2/3 Γ‐X, there are direct transitions with different bandgaps (i.e., non‐degenerate valleys) at these two points in reciprocal space, which have raised interest in SnS as a material for valleytronics.^[^ ^24^, ^25^ ^]^ We refer to them as X‐ and Y‐valleys. Calculations of the effective band structures of SnSe_1‐x_S_x_ alloys (see Experimental Section; Figure S9, Supporting Information) show the same features, i.e., an indirect fundamental bandgap and the X‐ and Y‐valleys across the entire range of compositions (Figure 6b–h). The primary effect of alloying is a systematic shift in the band‐edge energies, which are seen in plots showing small portions of *k*‐space around these special points as a function of composition, *x* (Figure 6i,j). The valence band maxima generally shift to higher energy while the conduction band minima drop with decreasing sulfur content. These combined shifts translate into a linear increase in the direct gaps at the X‐ and Y‐valleys, as well as of the fundamental bandgap, *E*_g_(*x*), with increasing sulfur content *x* in the alloys.

**Figure 7:**
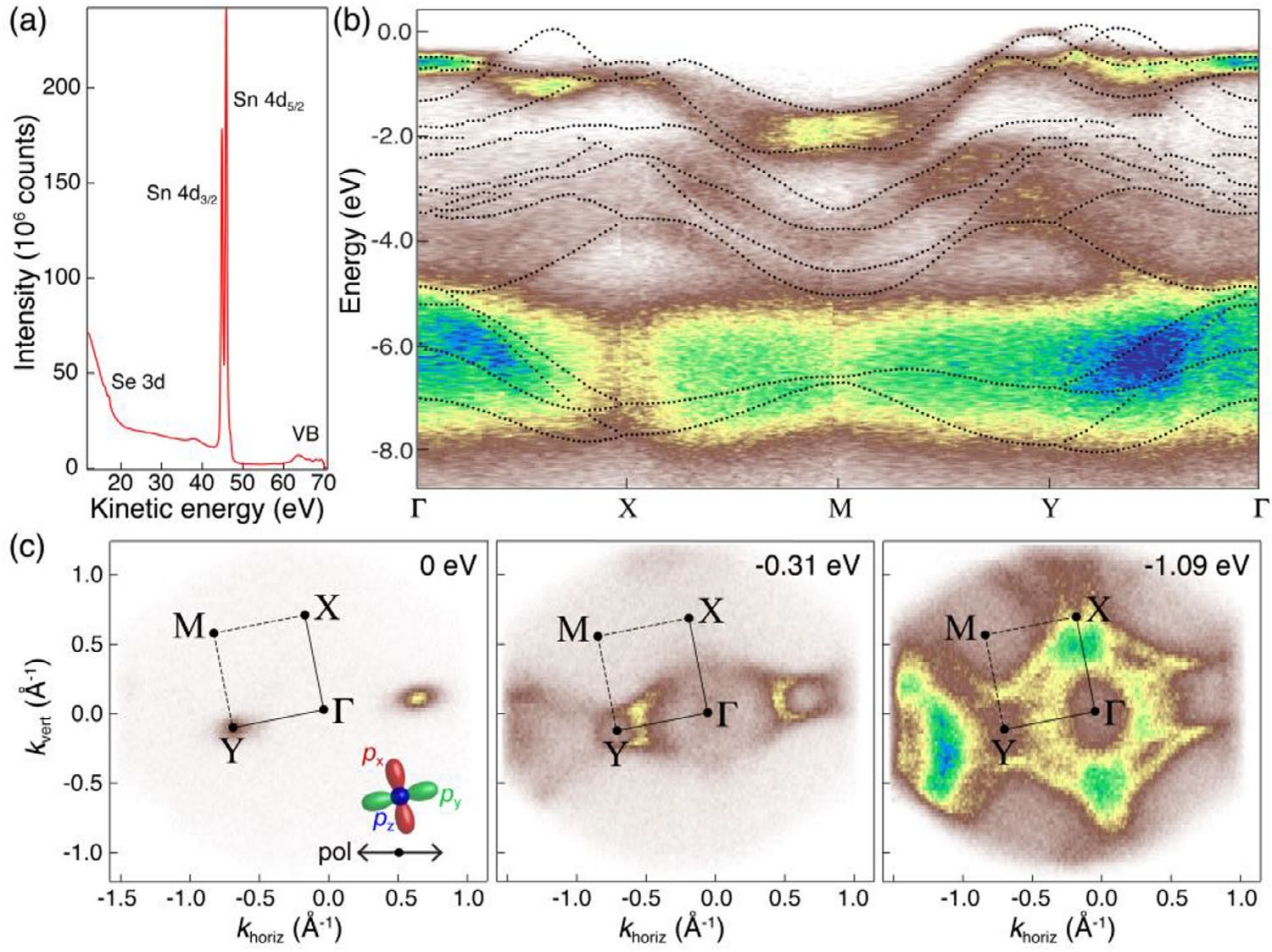
Synchrotron photoelectron spectroscopy of a SnSe_1‐x_S_x_ alloy flake. a) Photoelectron spectrum of a SnSe_1‐x_S_x_ alloy flake (*x* = 0.65), obtained with soft X‐rays (energy hν = 74 eV), showing shallow Se 3d and Sn 4d core levels, as well as intensity from the valence band (VB). b) Valence band structure of the SnSe_0.35_S_0.65_ alloy along high symmetry directions, determined by angle‐resolved photoelectron spectroscopy (ARPES). Dots represent the calculated effective band structure of an alloy with *x* = 0.625 (see Figure 6). c) Constant energy maps obtained at the valence band maximum (VBM) at Y, as well as 0.31 and 1.09 eV below the VBM. Horizontal arrow: Polarization of the incident soft X‐ray beam. Colored lobes represent the *p*_x_, *p*_y_, and *p*_z_ orbitals.

Nano‐ARPES was measured on individual SnSe_1‐x_S_x_ alloy flakes to validate the calculations and obtain further insight into the anisotropic electronic structure of the monochalcogenide alloys. **Figure** **7** summarizes the results of these experiments for the example of an SnSe_0.35_S_0.65_ alloy. Soft X‐ray synchrotron radiation with photon energy hν = 74 eV, focused into a submicrometer spot, was used to excite photoelectrons from single SnSe_1‐x_S_x_ flakes (see Experimental Section). Angle‐integrated photoelectron spectra show shallow Se 3*d* and Sn 4*d* core levels along with intensity originating from the valence band (Figure 7a). Figure 7b compares the measured dispersion of the valence bands, plotted along high‐symmetry directions in the Brillouin zone (see Figure 7c), with the calculated effective band structure of an alloy of the same composition. Overall, the measurements show a close correspondence to the calculated band structure. In particular, the valence band maximum at Y and the dispersion of most of the occupied bands closely follow the calculations. An exception is the valley along the Γ‐X line (i.e., the X‐valley), for which our ARPES measurements show no detectable intensity. Constant energy maps, presented in Figure 7c–e, also reflect the main features of the band structure. Notably, the maps at the valence band maximum (VBM, Figure 7c) and at somewhat lower energy (Figure 7d) prominently show the Y‐valley band edge but no detectable intensity from the X‐valley maximum. The bands immediately (0.5–1.5 eV) below the VBM have high intensity across most of the Brillouin zone. The lower‐lying valence bands (2–5 eV below the VBM) appear most intense near the Y‐point, but have lower intensity at M and are nearly absent along the Γ‐X line.

The prominence of the Y‐valley and absence of X‐valley signal in our ARPES measurement can be explained via the orbital character of the valence band edges and polarization of the exciting X‐ray beam. A theoretical analysis of the orbital contributions, shown in Figures S10 and S11 (Supporting Information) for SnS, reveals the major characteristics of valence‐ and conduction bands of the tin monochalcogenides. The conduction bands have mainly Sn‐*p* character, whereas the chalcogen (S) *p*‐orbitals dominate the valence bands. The breakdown into *p*_x_, *p*_y_, and *p*_z_ contributions reflects the pronounced in‐plane anisotropy of the tin monochalcogenides. In particular, the X‐valley VBM has mostly S‐*p*_x_ character while the Y‐valley maximum is dominated by S‐*p*_y_ orbitals. Photon polarization selection rules (i.e., the matrix element effect) stipulate that the signal from *p* orbitals is maximized for (s‐polarized) incident light with polarization along their principal axis.^[^ ^39^, ^40^ ^]^ In our case, the incident light was linearly polarized as shown in Figure 7c (see Figure S12 (Supporting Information) for an illustration of the measurement geometry), i.e., the polarization in the laboratory frame aligned closely with *p*_y_ and was nearly perpendicular to *p*_x_, which explains the large intensity of the Y‐valley and absence of signal from the X‐valley (Figure 7b,c).

The valence band edges at the X‐ and Y‐valleys have primarily *p*_x_ and *p*_y_ orbital character, but comparison with theory shows different orbital makeup of the lower‐lying valence bands (Figure S13, Supporting Information). All bands near the Y‐point have strong *p*_y_ contributions, but the bands at ≈1 eV below the VBM throughout the Brillouin zone, as well as the lower bands near the M‐point, also have significant *p*_z_ character. Overall, the ARPES results reflect the pronounced anisotropy of the electronic structure of the monochalcogenide alloys, which results from the strongly anisotropic bonding of this class of layered semiconductors.

**Figure 8:**
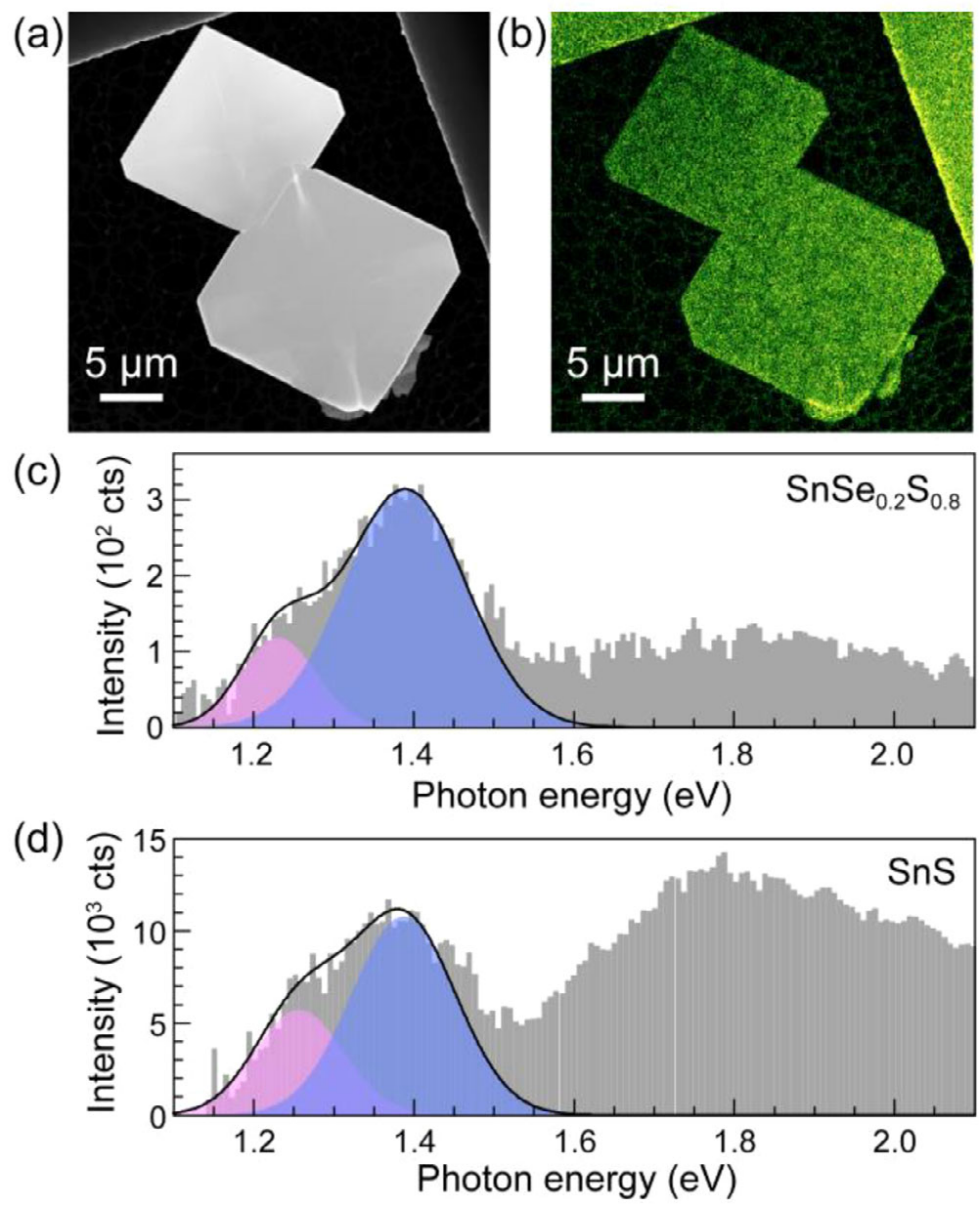
Cathodoluminescence spectroscopy of individual SnSe_1‐x_S_x_ alloy flakes. a) STEM image of two characteristic SnSe_1‐x_S_x_ alloy flakes (*x* = 0.80). b) Panchromatic STEM‐CL map of the two flakes shown in (a). c) STEM‐CL spectrum obtained on a SnSe_0.2_S_0.8_ alloy flake. d) STEM‐CL spectrum from a SnS flake. Shaded areas in (c) and (d) represent Gaussian fits to the two low‐energy peaks associated with the X‐valley (pink) and Y‐valley (blue) emission in the monochalcogenides. Black line: sum of both fits.

While in our ARPES measurements the X‐valley is suppressed due to selection rules, other experiments clearly show signatures of both the X‐ and Y‐valley transitions, which together with the computed band structures (Figure 6) confirm that SnSe_1‐x_S_x_ alloys across the entire composition range retain their unusual valley configuration, interesting for valleytronics. Specifically, we used cathodoluminescence spectroscopy in scanning transmission electron microscopy (STEM‐CL) to probe the direct optical transitions across the (X, Y) valleys in SnSe_1‐x_S_x_ alloy flakes. Such measurements previously confirmed the direct transitions in the two valleys for pure SnS and demonstrated the valley‐selective quenching of the luminescence in SnS‐GeS van der Waals stacks.^[^ ^25^ ^]^ Due to the limitation of the CL detector to photon energies above the Si bandgap, the measurements were carried out on S‐rich alloys whose direct transitions yield emissions at photon energies above 1.2 eV. **Figure** **8** shows the example of SnSe_0.2_S_0.8_. Figure 8a is a HAADF‐STEM image of two alloy flakes with this composition; the corresponding panchromatic CL map demonstrates bright light emission from the flakes (Figure 8b). A STEM‐CL spectrum, obtained near the center of one of the flakes, is shown in Figure 8c. The low‐energy light emission can be fitted to two distinct peaks, centered at photon energies of 1.23 and 1.39 eV, respectively. To ascertain the origin of these peaks, we compare with the emission from a pure SnS flake (Figure 8d). Here, a similar peak shape is observed that can be deconvoluted into two components centered at 1.26 and 1.40 eV, respectively, due to direct optical transitions at the X‐ and Y‐valley of SnS.^[^ ^4^, ^25^, ^41^, ^42^ ^]^ We conclude that the emission from the SnSe_1‐x_S_x_ alloy shows contributions from the X‐ and Y‐valley direct transitions, albeit red‐shifted compared to SnS, consistent with the bandgap narrowing due to alloying with Se (see Figure 6). The combined STEM‐CL and computational results confirm that SnSe_1‐x_S_x_ alloys across the full range of compositions retain the unique band configuration comprising two non‐degenerate valleys along orthogonal *k*‐space directions. Hence, the alloys offer the opportunity to continuously tune the transition energies across the two valleys, supporting their application as unconventional semiconductors for valleytronics.

## 3 Conclusion

In conclusion, we applied combined ab‐initio calculations and measurements to determine important properties of SnSe_1‐x_S_x_ alloys, intermediate between the van der Waals semiconductors SnS and SnSe. The experiments were enabled by a vapor transport growth process, in which systematic changes to a mixed SnS‐SnSe powder precursor in consecutive growth runs are harnessed to access series of SnSe_1‐x_S_x_ alloy flakes with different composition. Performed on mica van der Waals substrates, the growth consistently yields large (≈20 µm edge length) single‐crystalline flakes whose structure, electronic, and vibrational properties interpolate between those of the endpoint compounds. While calculations predict random alloys to be stable across the entire composition range, cross‐sectional STEM imaging shows consistent variations in S:Se ratio between adjacent layers in the monochalcogenide unit cell, which are likely due to oscillations in the abundance of adsorbed sulfide and selenide precursor molecules on the surface of the growing flakes, combined with a pronounced layer‐by‐layer growth mode. The endpoint compounds SnS and SnSe have attracted special interest due to their anisotropic structure and properties. Calculations and measurements demonstrate that these characteristics persist across the SnSe_1‐x_S_x_ alloys, notably in the form of two non‐degenerate direct transitions (valleys) along the X and Y in‐plane directions in reciprocal space. The band‐edges at the valleys, as well as the overall band structure of the alloys, systematically change between SnS and SnSe. ARPES measurements of the valence band structure for a representative alloy generally agree well with calculations, and excitation by polarized light gives rise to pronounced matrix element effects due to the anisotropy of the electronic structure. Luminescence measurements on single alloy flakes, finally, directly demonstrate X‐ and Y‐valley transitions in the alloys. Given the broad interest in tin monochalcogenides for applications such as thermoelectrics, optoelectronics, and valleytronics – so far pursued mostly in the endpoint compounds SnS and SnSe – and the ability to tune structure and electronic properties by alloying, the present study on the synthesis and properties of high‐quality SnSe_1‐x_S_x_ alloy flakes opens up avenues for materials design in support of both existing and emerging applications of layered monochalcogenide semiconductors.

## 4 Experimental Section

### Alloy Growth

SnSe_1−_ *_x_* S*_x_* flakes were grown by vapor transport using mixtures of SnS (99.99%; Sigma Aldrich) and SnSe (99.999%; ALB Materials) powder precursors. The growth was performed in a pumped 2‐inch quartz tube reactor with a single temperature‐controlled zone (MTI model OTF‐1200X). SnS and SnSe powders were mixed and placed in the same quartz boat in the center of the evaporation zone, which was then heated to 580 °C. Substrates consisted of freshly exfoliated mica crystals (Ted Pella), supported by a molybdenum plate and placed ≈10 cm downstream from the source boat. During growth, a flow of Ar carrier gas (99.9999%, Matheson) was maintained at 60 standard cubic centimeters per minute and a pressure of 20 mTorr. The growth duration was typically 30 minutes at 580 °C. After completion of the growth, the reactor was cooled naturally to room temperature.

### Optical Measurements

Polarized optical microscopy was performed in reflection geometry using an upright microscope (Olympus BX53) equipped with a fixed incident‐light polarizer and adjustable reflected‐light analyzer, a 100× objective, and a high‐resolution (12.37 megapixel; Olympus DP75) scientific camera. Micro‐Raman spectroscopy was performed in air using a Raman microscope (Horiba Xplora plus) with a 100× objective, 532 nm excitation wavelength, and 16.8 µW laser power. Raman spectra were measured using a 300 µm pinhole at ≈0.5 µm spatial resolution. Optical reflectance spectra were recorded on samples with a high areal coverage of alloy flakes on the mica growth substrates using a Perkin Elmer Lambda 750 spectrophotometer equipped with a 100 mm integrating sphere and InGaAs detector.

### Electron Microscopy

Structure and morphology of the SnSe_1−_ *_x_* S*_x_* alloy flakes were investigated by TEM and nanobeam electron diffraction in an FEI Talos F200X microscope operated at 200 kV on crystals transferred onto lacey carbon grids. Samples for cross‐sectional (S)TEM were prepared using the in situ lift‐out method utilizing a Hitachi NB5000 FIB and a FEI Helios G5 UX Dual Beam FIB/SEM with final Ga^+^ milling performed at 2 keV. Composition measurements were performed using energy‐dispersive X‐ray spectroscopy (EDS) in an FEI Talos F200X microscope at 200 kV. Atomic‐resolution HAADF‐STEM imaging and electron energy loss spectroscopy (EELS) were performed on cross‐sectional samples in a JEOL ARM200CF microscope operated at 200 kV. Atomic positions were determined from raw HAADF‐STEM images as described previously.^[^ ^43^ ^]^ STEM‐cathodoluminescence (STEM‐CL) was performed using a Gatan Vulcan CL holder at 200 keV electron energy and an incident beam current of ≈400 pA. Panchromatic CL maps (512 × 512 pixels, 1.28 ms per pixel) were acquired by scanning the focused electron beam and recording the emitted light intensity over a broad wavelength range (400–1000 nm). STEM‐CL spectra were acquired by placing the exciting electron beam near the center of SnSe_1‐x_S_x_ and SnS flakes, collecting the emitted light into a spectrograph, and detection using a Si CCD array detector (integration time: 10s).

### Angle‐Resolved Photoelectron Spectroscopy

ARPES was performed at the Spectromicroscopy beamline of the Elettra light source, with linearly polarized radiation of 74 eV focused to a ≈0.6‐µm diameter spot by a Schwarzschild objective and incident at 52° with respect to the sample normal (see Figure S12, Supporting Information).^[^ ^44^ ^]^ Photoelectons originating from the flakes were acquired from a cone of 35° aperture with a deflector type A1 hemispherical analyzer (MB Scientific). The overall energy and angular resolution were ≈80 meV and ≈0.5°, respectively. Atomically clean and ordered surfaces of the flakes were obtained by repeated cycles of sputtering and annealing in a preparation chamber with a base pressure of 3 × 10^−10^ mbar, directly connected to the main measurement station (base pressure: 2 × 10^−10^ mbar) where the measurements were performed at room temperature.

### Computations

All DFT calculations were carried out using the VASP package.^[^ ^45^, ^46^ ^]^ The study used a plane‐wave cutoff of 500 eV and a *k*‐point mesh density corresponding to a 12 × 8 × 4 grid in the bulk unit cell or better. Based on benchmarking of the lattice constants (Table S1, Supporting Information), the rev‐vdW‐DF2 functional of Hamada^[^ ^47^ ^]^ was adopted, which yielded close agreement with experimental values for both SnS and SnSe.

To study S/Se alloying, a cluster expansion (CE) was first built using the ATAT package.^[^ ^48^, ^49^ ^]^ The CE was obtained by fitting to 70 automatically generated structures. The good quality of the CE is demonstrated by the cross‐validation score of 0.41 meV per S/Se site and the correlation plot shown in Figure S1b (Supporting Information). The mixing energies are low (Figure S1a, Supporting Information), which leads to perfect miscibility at temperatures below 300K (Figure S1c,d, Supporting Information).

As the CE indicated that mixing of S and Se is preferred, the alloy properties were evaluated using 4 × 4 × 1 supercell special quasi‐random structures (SQS), also generated using the ATAT package.^[^ ^50^ ^]^ The effective band structures (EBS) were extracted using the BandUP package.^[^ ^51^ ^]^ EBS plots were created as follows. The study considered small energy intervals (width of 10 meV) as in conventional density‐of‐states plots. Each state of the supercell that falls within one of these energy intervals is projected to plane waves corresponding to the primitive cell *k*‐points. The effective band structure “value” at that *k*‐point and energy interval is increased by the corresponding projection coefficient. Thus, each state can project to many primitive cell *k*‐points with non‐zero coefficient if translational symmetry is broken due to alloying, and thus the EBS shown in Figure 5 appears necessarily “blurred”.

## Conflict of Interest

The authors declare no conflict of interest.

## Supporting information



Supporting Information

## Data Availability

The data that support the findings of this study are available from the corresponding author upon reasonable request.
