# Supplementary material for: SnSe1‐xSx Alloys: Anisotropic Van der Waals Semiconductors with Tunable Bandgaps
Source: Small. 2025 Nov 27;22(4):e08578. doi: 10.1002/smll.202508578 (PMC12809197; doi:10.1002/smll.202508578)
Supplement: Supplementary file 1 — Supporting Information [file SMLL-22-e08578-s001.docx]

**Supporting Information**

**SnSe_1-x_S_x_ Alloys: Anisotropic van der Waals Semiconductors with Tunable Bandgaps**

Peter Sutter,^1,*^ Alexei Barinov,^2^ Hannu-Pekka Komsa,^3^ Pramod Ghimire,^4^ Lijun Wu,^5^ Yimei Zhu,^5^ Kim Kisslinger,^6^ and Eli Sutter^4,7,*^

^1^Department of Electrical & Computer Engineering, University of Nebraska-Lincoln, Lincoln, Nebraska 68588, United States

^2^Elettra Sincrotrone Trieste SCpA, 34149 Basovizza, Trieste, Italy

^3^Microelectronics Research Unit, University of Oulu, FI-90014 Oulu, Finland

^4^Department of Mechanical & Materials Engineering, University of Nebraska-Lincoln, Lincoln, Nebraska 68588, United States

^5^Condensed Matter Physics and Materials Science Department, Brookhaven National Laboratory, Upton, New York 11973, United States

^6^Center for Functional Nanomaterials, Brookhaven National Laboratory, Upton, New York 11973, United States

^7^Nebraska Center for Materials & Nanoscience, University of Nebraska-Lincoln, Lincoln, Nebraska 68588, United States

^*^Corresponding authors, e-mail: psutter@unl.edu, esutter@unl.edu


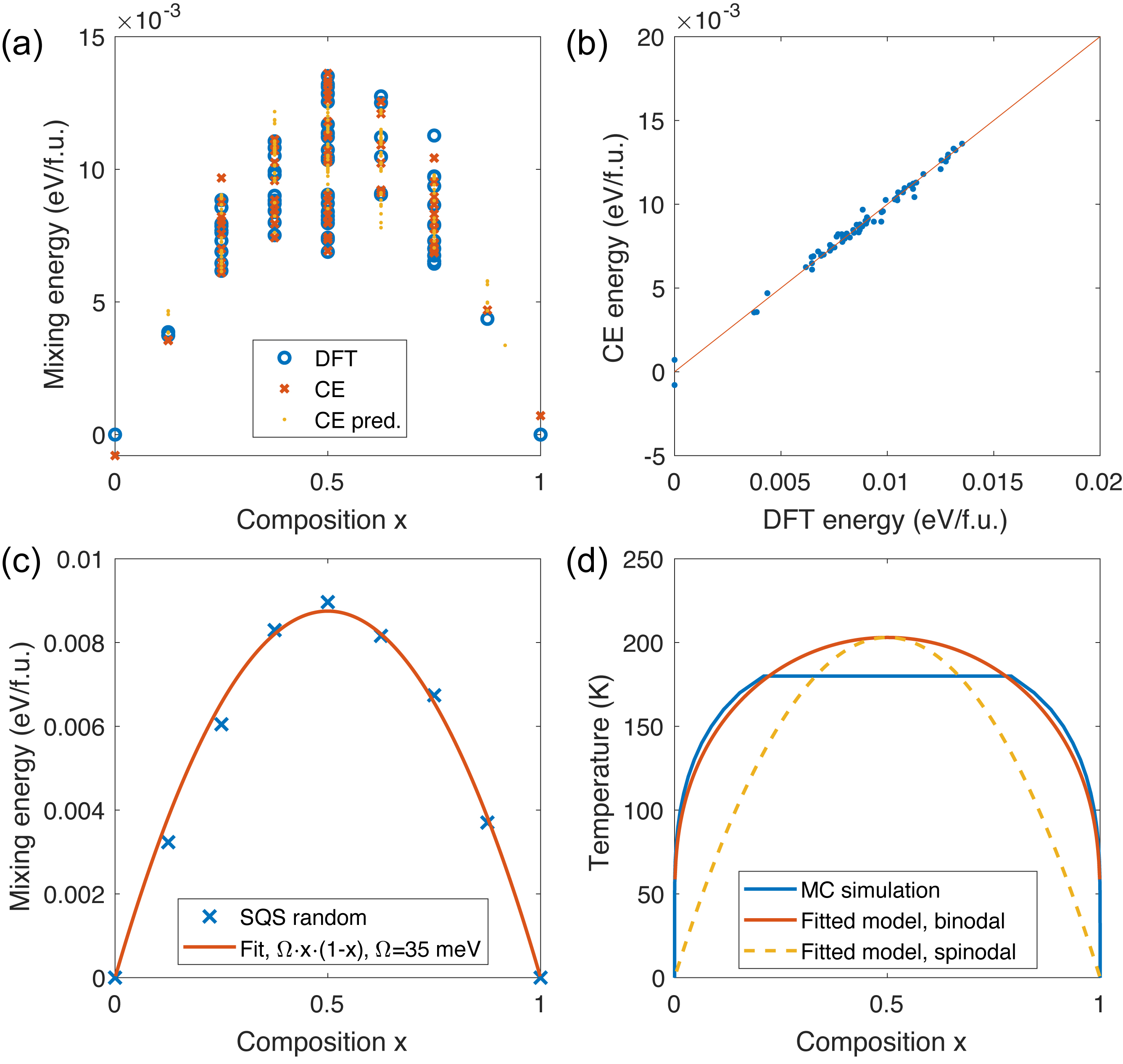


**Figure S1. Mixing energies of SnSe_1-x_S_x_ alloys obtained using the Alloy Theoretic Automated Toolkit (ATAT). (a)** Mixing energies as a function of composition. **(b)** Comparison of the mixing energy (per formula unit, f.u.) obtained by density functional theory (DFT) and by cluster expansion (CE). **(c)** Fit of a parabolic energy model to the mixing energies from SQS. **(d)** Phase diagram of SnSe_1-x_S_x_ alloys obtained by Monte Carlo (MC) simulation and *via* the parabolic model.


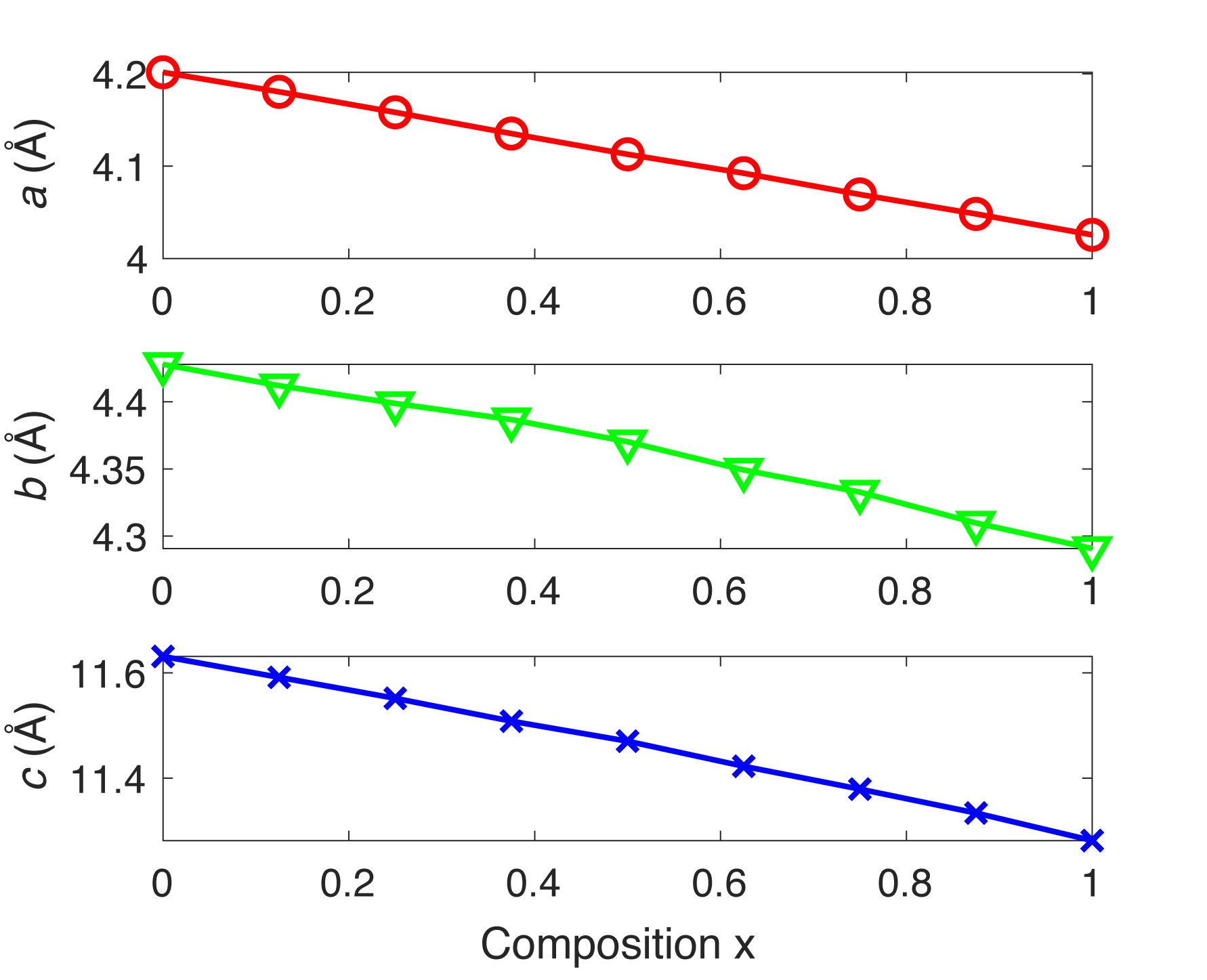


**Figure S2. Calculated lattice constants of SnSe_1-x_S_x_ alloys.** Computations used the rev-vdW-DF2 functional, which yields the closest match between calculated and measured in-plane lattice parameters (*a*, *b*) for the endpoint compounds SnS and SnSe (see Table S1).


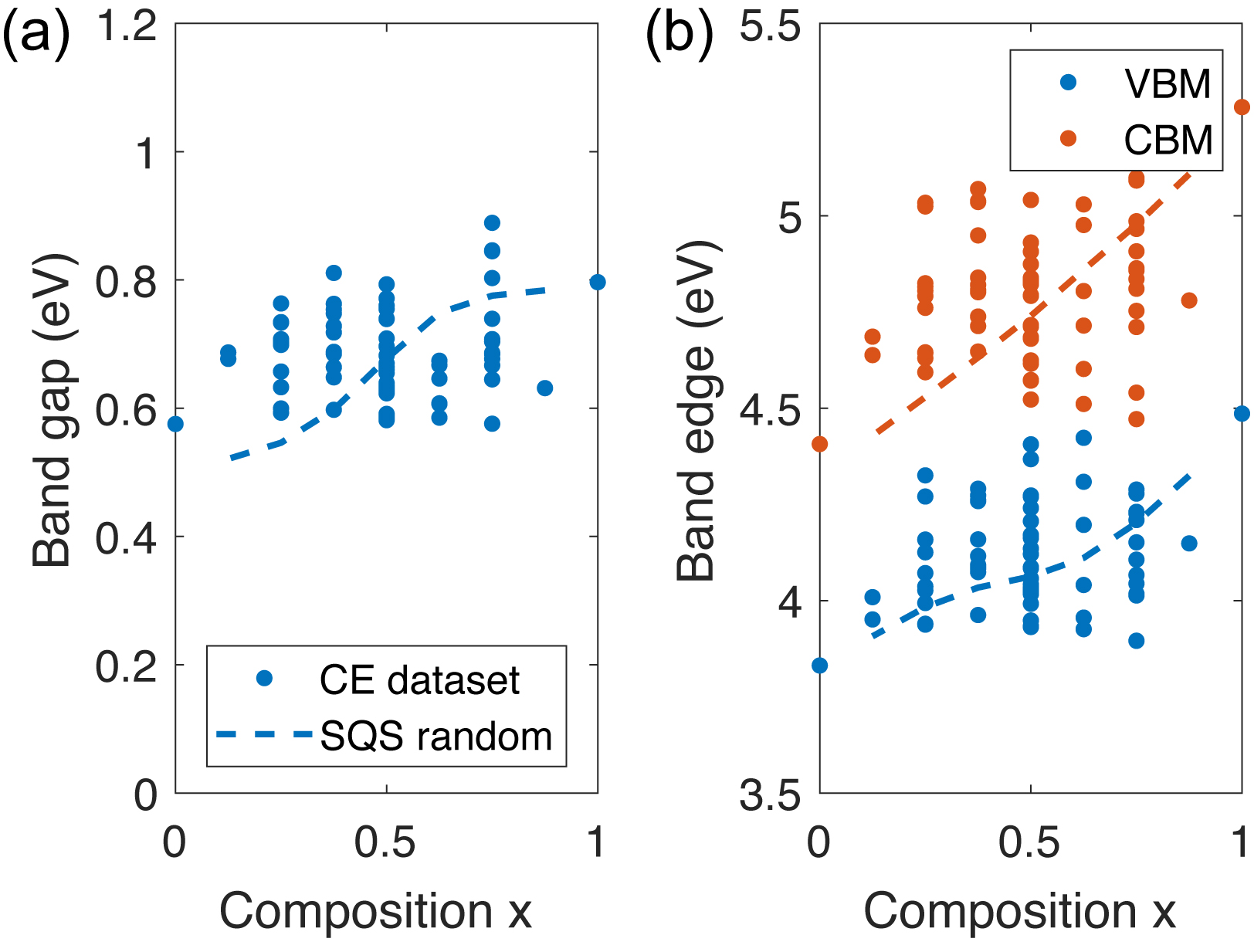


**Figure S3. Composition dependent bandgap and band edges of SnSe_1-x_S_x_ alloys. (a)** Bandgap. **(b)** Energies of the valence band maximum (VBM) and conduction band minimum (CBM).

**
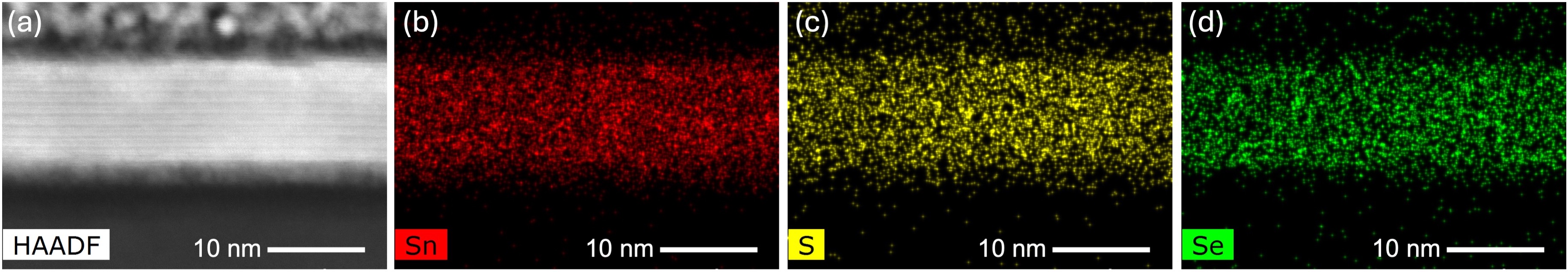
**

**Figure S4. Cross-sectional EDS mapping of a SnSe_1-x_S_x_ alloy flake. (a)** HAADF-STEM image of the few-layer SnSe_0.36_S_0.64_ alloy flake. **(b) – (d)** EDS maps showing the distribution of Sn (b), S (c), and Se (d) across the flake.

**
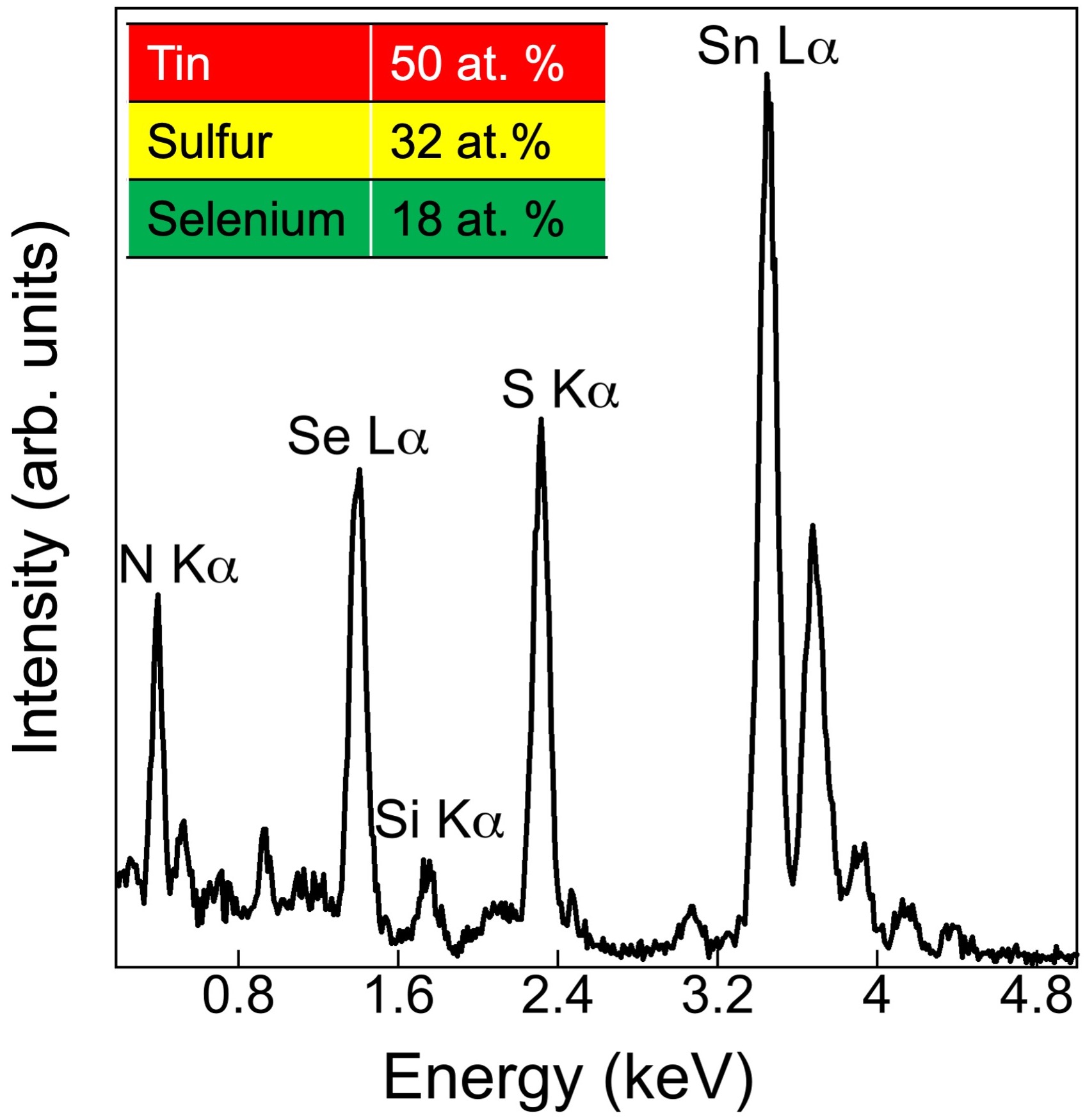
**

**Figure S5. Quantification of the EDS spectrum of a SnSe_1-x_S_x_ alloy flake.** EDS spectrum acquired in STEM on a cross-sectional sample of a SnSe_0.36_S_0.64_ alloy flake (see Figure S4).

**
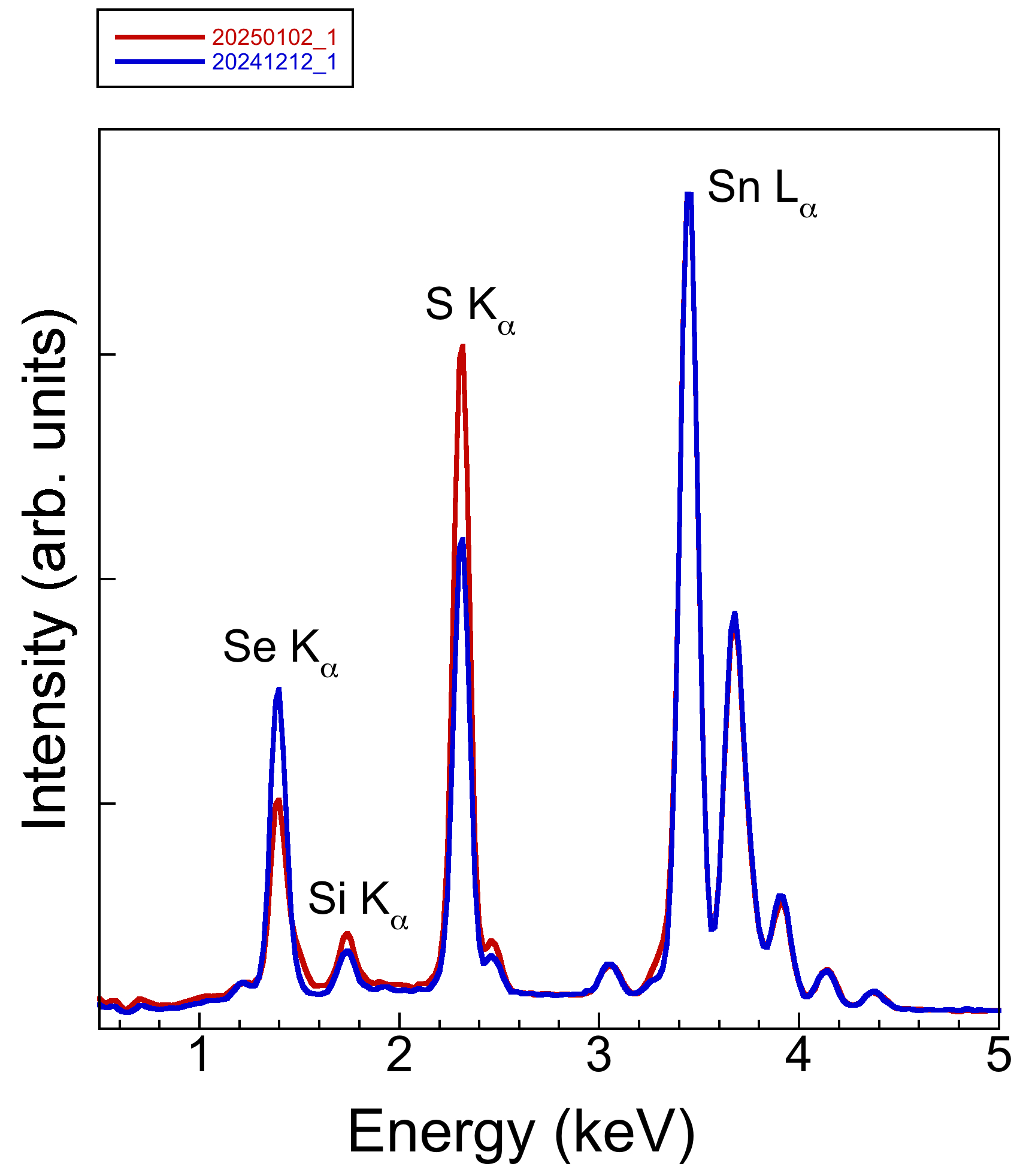
**

**Figure S6. EDS spectra of two SnSe_1-x_S_x_ alloy flakes with different composition.** Spectra acquired in SEM on plan-view samples, quantified to alloy compositions SnSe_0.20_S_0.80_ (red) and SnSe_0.36_S_0.64_ (blue).


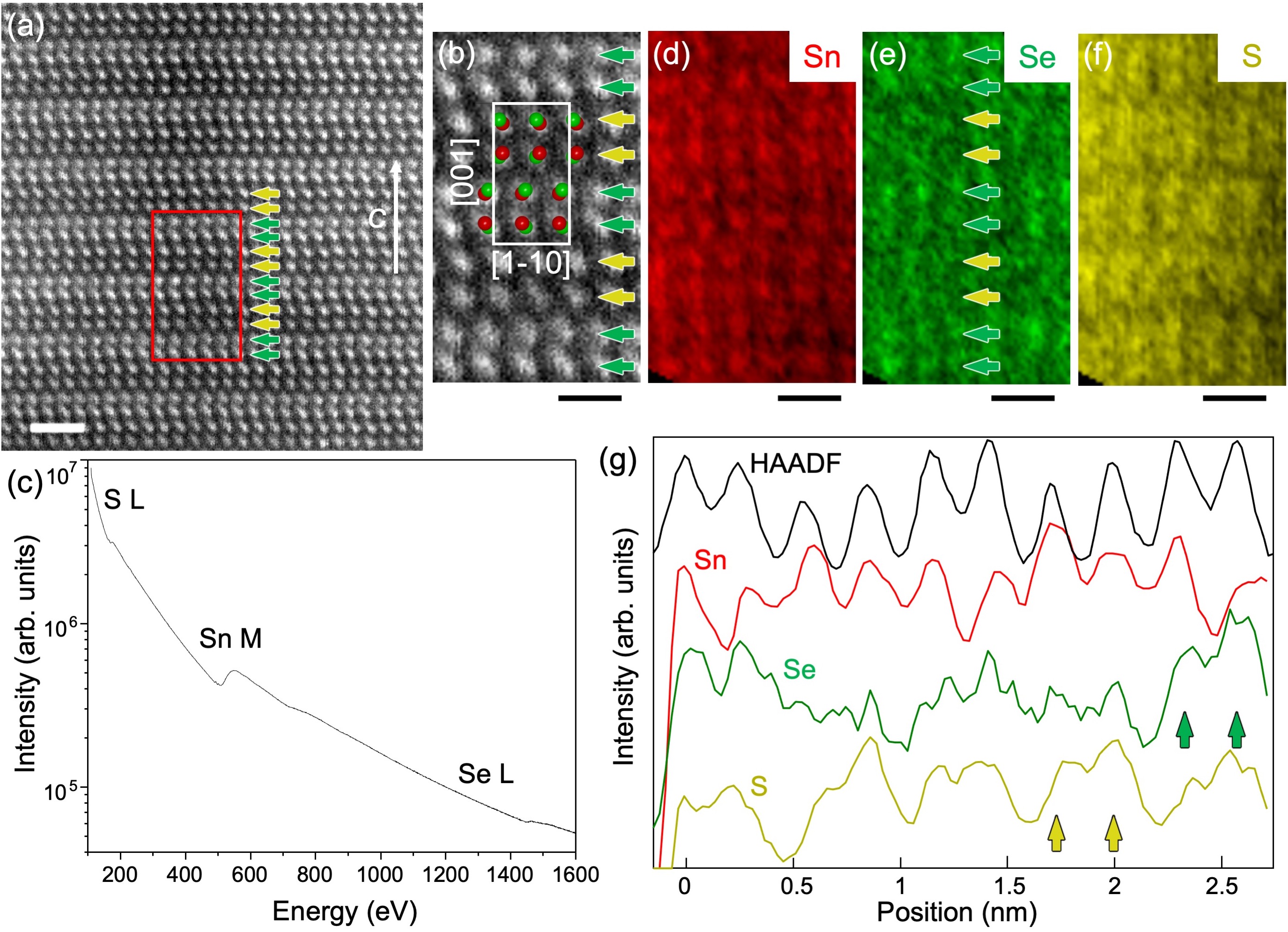


**Figure S7. STEM-EELS analysis of the chalcogen distribution in alternating layers. (a)** HAADF-STEM image of an SnSe_1-x_S_x_ alloy flake. Scale bar: 1 nm. **(b)** Magnified image from the area marked by the rectangle in (a). The overlay shows the [110] projection of the SnSeS structure model. A 2D electron energy loss spectrum (EELS) image is acquired from this area for elemental mapping in (d) – (f). Scale bar: 0.5 nm. **(c)** EELS spectrum from the area shown in (b). The intensity is shown on a logarithmic scale. **(d) – (f)** Integrated EELS signal intensity of Sn L (d), Se M (e), and S L edges (f) after background subtraction. Scale bar: 0.5 nm. **(g)** Intensity profiles from the HAADF-STEM image (b, black) and the EELS maps (d: red, e: green, f: yellow) obtained by integrating the intensity horizontally in (b) and (d) – (f). The pairs of atomic columns marked by green arrows have high HAADF-STEM image intensity as well as large Se and weak S signal, while the columns marked by yellow arrows have lower HAADF-STEM intensity as well as smaller Se and larger S signal.


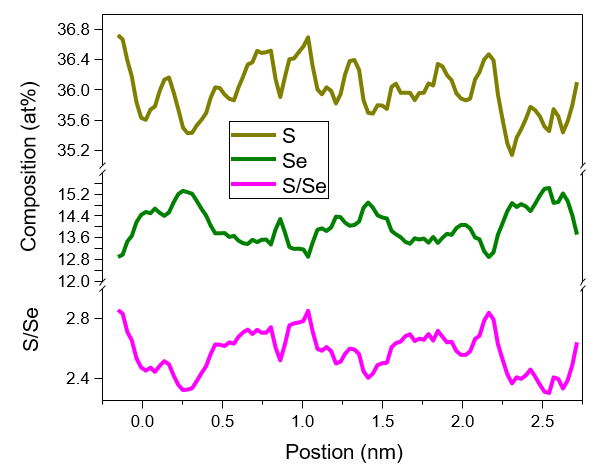


**Figure S8. Quantification of the S:Se ratio in consecutive layers.** Analysis of STEM-EELS of a SnSe_0.28_S_0.72_ alloy flake, showing a variation in the S:Se ratio between 2.3 and 2.8 in consecutive layers.


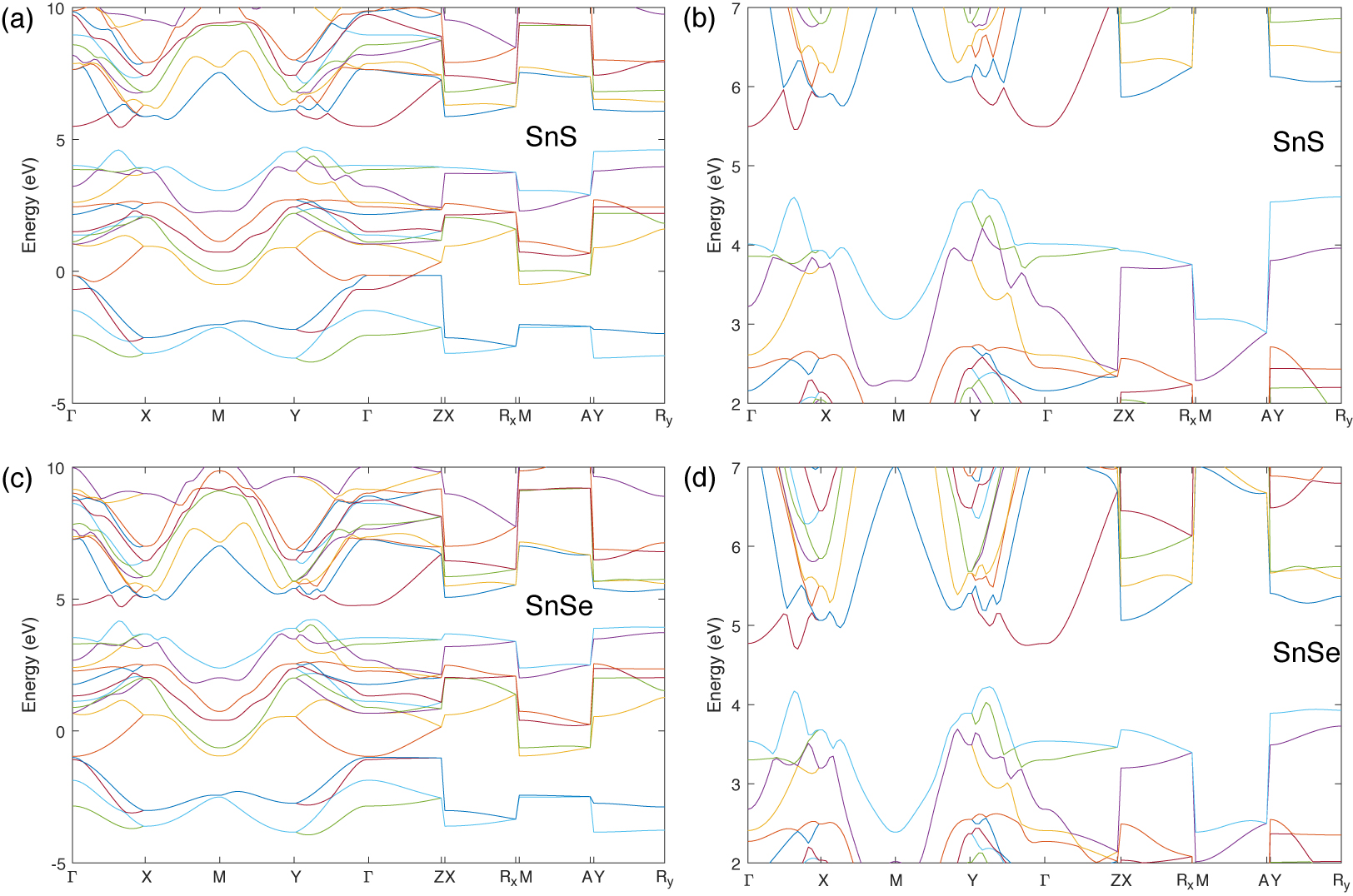


**Figure S9. Calculated band structures of SnS and SnSe. (a), (b)** Band structure of SnS. **(c), (d)** Band structure of SnSe.


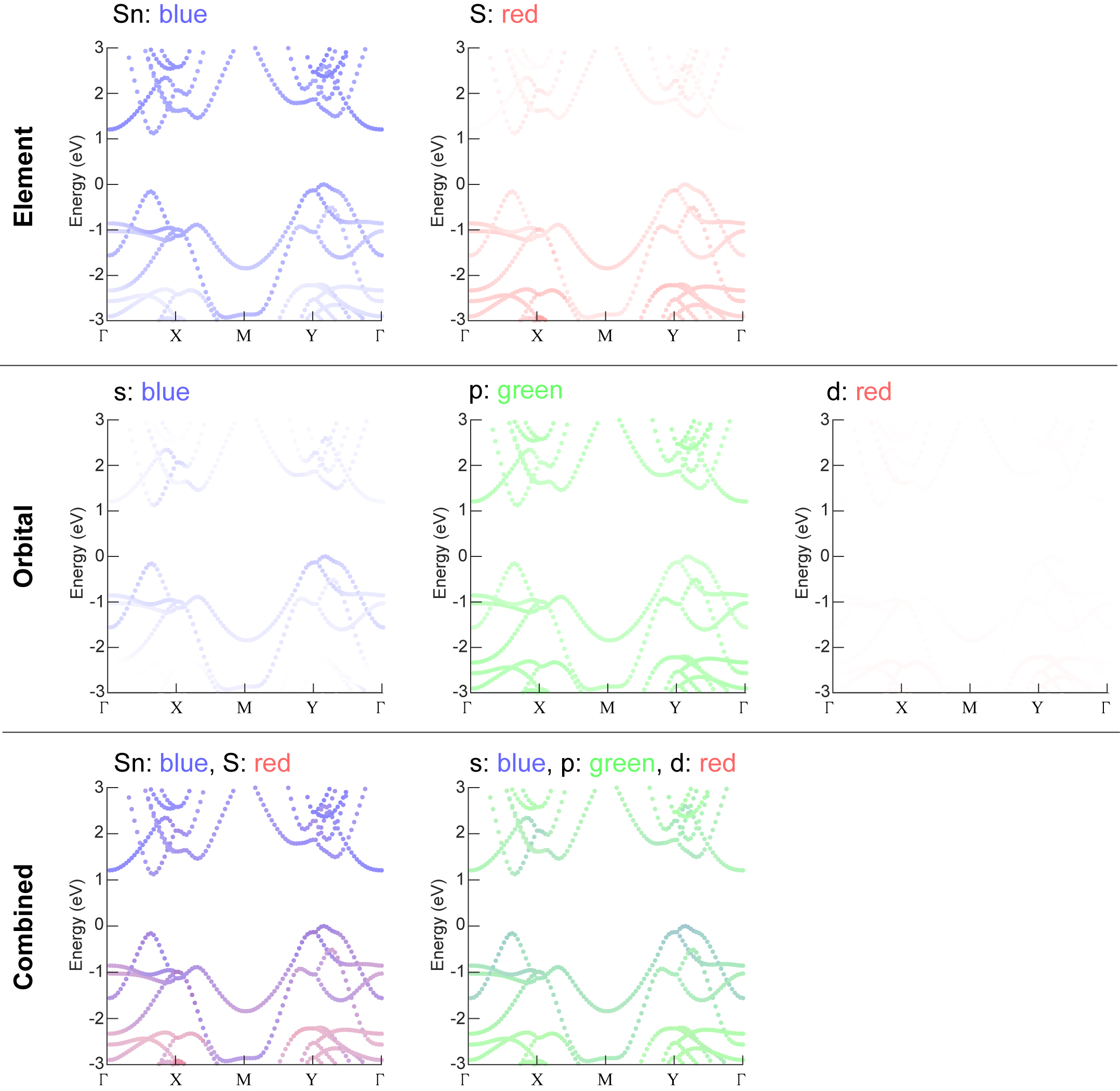


**Figure S10. Orbital character of the valence and conduction bands for the example of SnS.** The DFT analysis shows contributions of cations (Sn, blue) and anions (S, red) to valence and conduction bands (top row), the orbital character of the bands (*s*, blue; *p*, green; *d*, red; center row), and the combined picture (bottom row). Conclusion: The valence bands have mostly S *p*-orbital character, while the conduction bands are primarily determined by Sn *p*-orbitals.


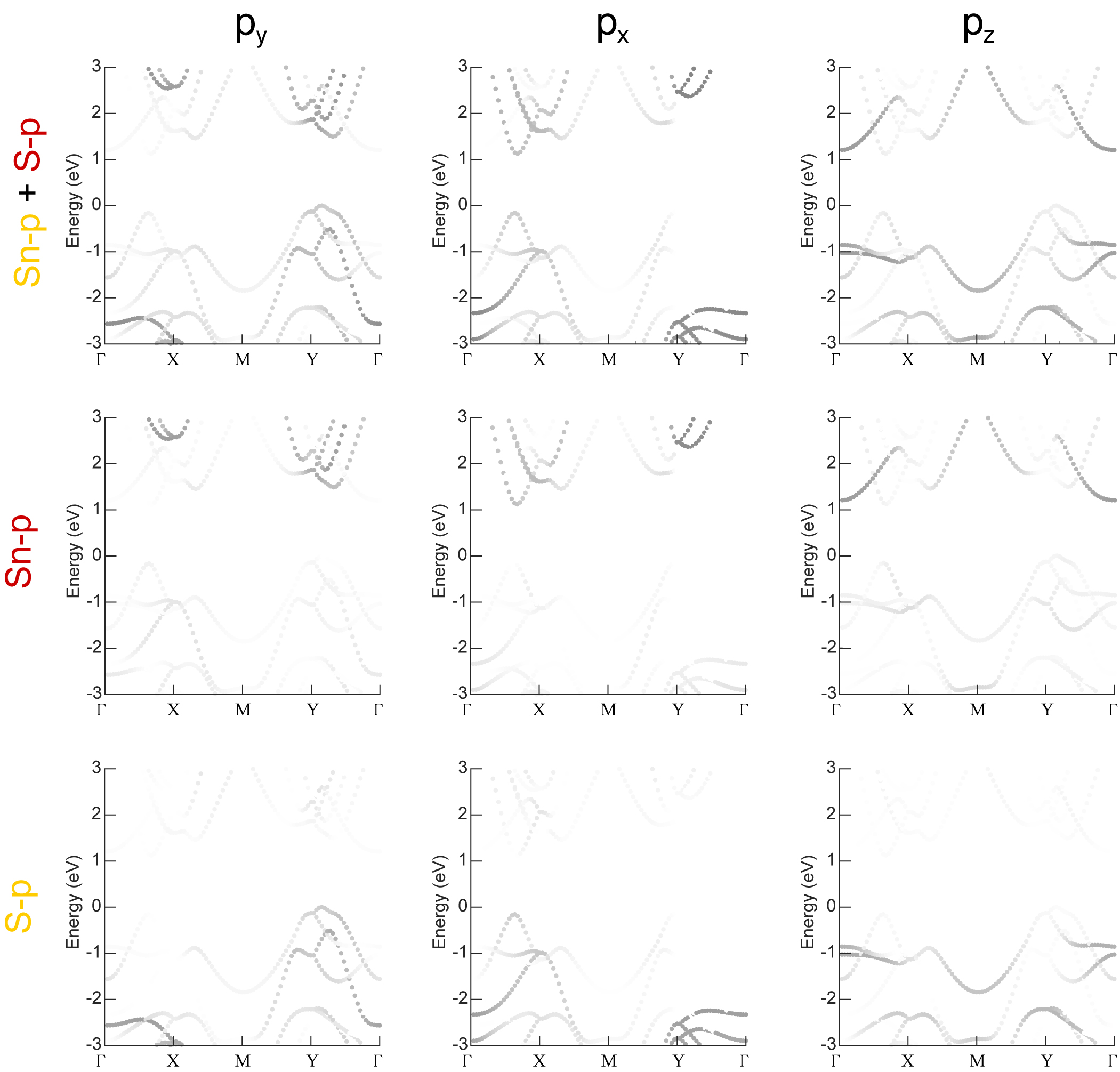


**Figure S11. Orbital character of the valence and conduction bands for the example of SnS.** The analysis shows the contributions of S *p*-orbitals (bottom row), Sn *p*-orbitals (middle row), and of S and Sn *p*-orbitals (top row), broken down into *p*_x_, *p*_y_, and *p*_z_ components (columns). Note that the conduction bands are mainly derived from Sn *p*-orbitals whereas the valence bands have mostly S *p*-orbital character. Gray level encodes the importance of the orbital contribution, ranging from white (0%) to black (100%).


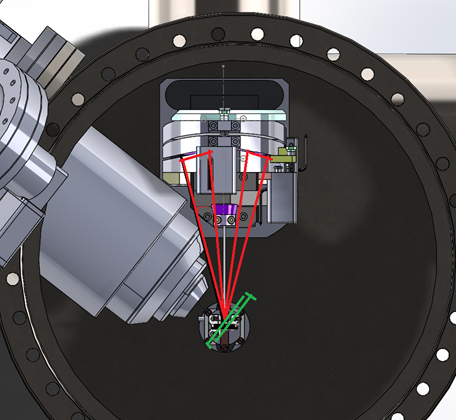


**Figure S12. Experimental geometry of synchrotron ARPES measurements.** The linearly polarized soft X-ray beam is incident from the top from a Schwarzschild objective (red lines), giving rise to a focused illumination cone on the sample surface (green). The sample is tilted by 52 degrees relative to the cone axis (thus providing a mixture of s-polarization and p-polarization of the incident light). The axis of the analyzer (left) is oriented along the sample normal, *i.e.*, the analyzer collects photoelectrons emitted perpendicular to the sample surface.


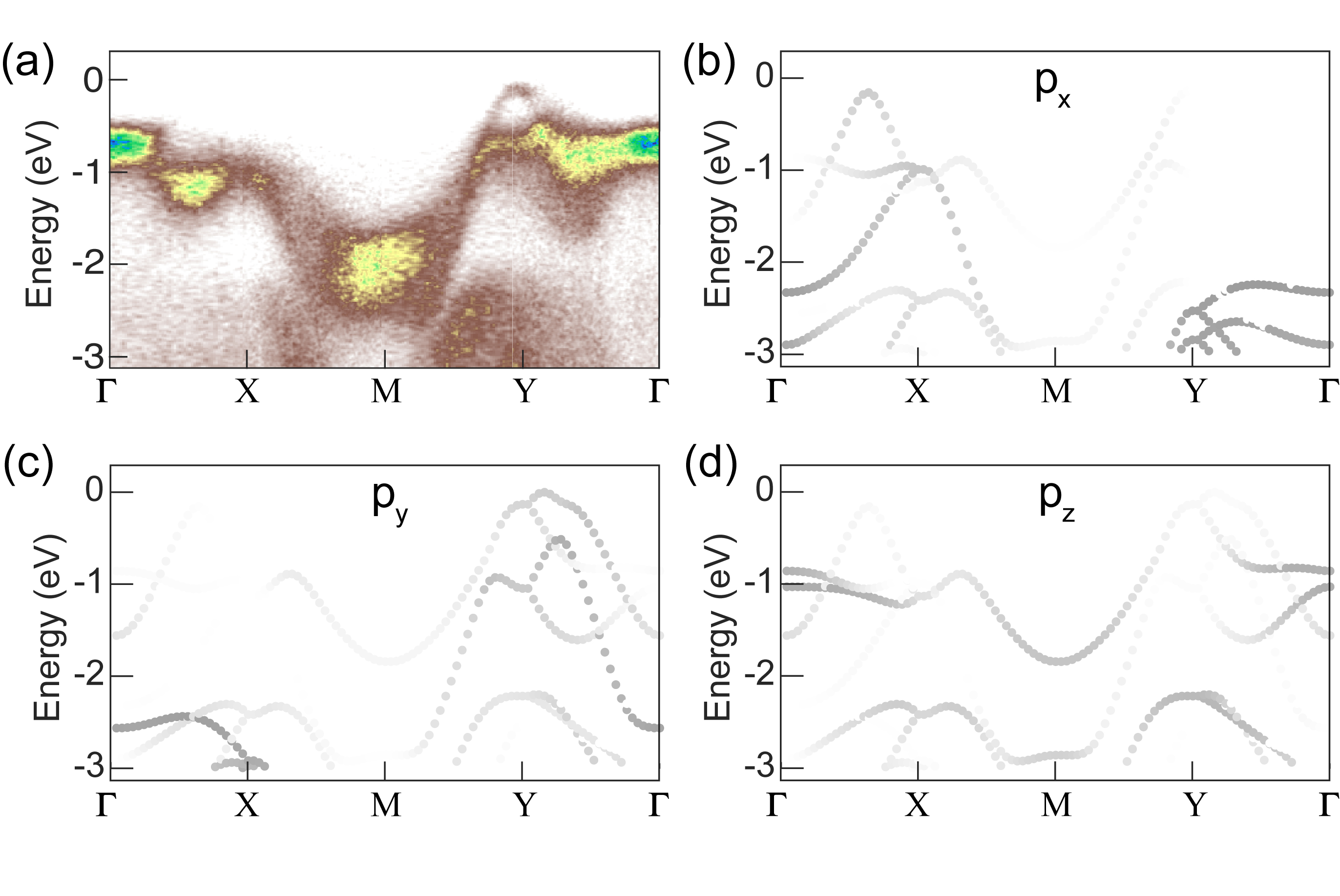


**Figure S13. ARPES spectrum near the valence band maximum (SnSe_0.35_S_0.65_) compared with SnS bands derived from sulfur p_x,y,z_ orbitals. (a)** ARPES band map within 3 eV of the valence band maximum. **(b) – (d)** Calculated bands of SnS decomposed into contributions from sulfur *p*_x_ (b), *p*_y_ (c), and *p*_z_ (d) orbitals.

**Table S1. Comparison of experimental SnS and SnSe lattice constants (measured by electron diffraction) with lattice parameters calculated with different functionals (all values in Å).**

|  | SnS | | | SnSe | | |
| --- | --- | --- | --- | --- | --- | --- |
|  | *a* | *b* | *c* | *a* | *b* | *c* |
| Expt. | 4.031 | 4.285 |  | 4.214 | 4.420 |  |
| LDA | 3.955 | 4.165 | 10.970 | 4.118 | 4.305 | 11.310 |
| PBE | 1.024 | 4.422 | 11.424 | 4.206 | 4.551 | 11.759 |
| PBE-D2 | 4.009 | 4.266 | 11.384 | 4.195 | 4.356 | 11.634 |
| rev-vdW-DF2 | 4.026 | 4.291 | 11.281 | 4.202 | 4.428 | 11.631 |
